# Developing Hypoimmunogenic Human iPSC‐Derived Oligodendrocyte Progenitor Cells as an Off‐The‐Shelf Cell Therapy for Myelin Disorders

**DOI:** 10.1002/advs.202206910

**Published:** 2023-06-04

**Authors:** Lizhao Feng, Jianfei Chao, Peng Ye, Qui Luong, Guoqiang Sun, Wei Liu, Qi Cui, Sergio Flores, Natasha Jackson, Afm Nazmul Hoque Shayento, Guihua Sun, Zhenqing Liu, Weidong Hu, Yanhong Shi

**Affiliations:** ^1^ Department of Neurodegenerative Diseases Beckman Research Institute of City of Hope 1500 E. Duarte Rd. Duarte CA 91010 USA; ^2^ Department of Immunology and Theranostics Beckman Research Institute of City of Hope 1500 E. Duarte Rd. Duarte CA 91010 USA

**Keywords:** allogeneic cell therapy, Canavan disease, demyelinating diseases, iPSCs, off‐the‐shelf cell therapy, oligodendrocyte progenitor cells (OPC), universal donor

## Abstract

Demyelinating disorders are among the most common and debilitating diseases in neurology. Canavan disease (CD) is a lethal demyelinating disease caused by mutation of the aspartoacylase (*ASPA*) gene, which leads to the accumulation of its substrate *N*‐acetyl‐l‐aspartate (NAA), and consequently demyelination and vacuolation in the brain. In this study, hypoimmunogenic human induced pluripotent stem cell (iPSC)‐derived oligodendrocyte progenitor cells (OPC) are developed from a healthy donor as an “off‐the‐shelf” cell therapy. Hypoimmunogenic iPSCs are generated through CRISPR/Cas9 editing of the human leukocyte antigen (HLA) molecules in healthy donor‐derived iPSCs and differentiated into OPCs. The OPCs are engrafted into the brains of CD (nur7) mice and exhibit widespread distribution in the brain. The engrafted OPCs mature into oligodendrocytes that express the endogenous wildtype *ASPA* gene. Consequently, the transplanted mice exhibit elevated human ASPA expression and enzymatic activity and reduced NAA level in the brain. The transplanted OPCs are able to rescue major pathological features of CD, including defective myelination, extensive vacuolation, and motor function deficits. Moreover, the hypoimmunogenic OPCs exhibit low immunogenicity both in vitro and in vivo. The hypoimmunogenic OPCs can be used as “off‐the‐shelf” universal donor cells to treat various CD patients and many other demyelinating disorders, especially autoimmune demyelinating diseases, such as multiple sclerosis.

## Graphical abstract

Canavan disease (CD) is a demyelinating disorder caused by mutation of the *ASPA* gene. Human hypoimmunogenic oligodendrocyte progenitor cells (OPCs) are generated with the wildtype *ASPA* gene. These cells exhibit low immunogenicity in vitro and in vivo. In a CD mouse model, the engrafted OPCs mature into oligodendrocytes to express endogenous ASPA, repair myelination, and rescue main pathologies of CD.

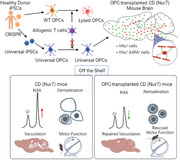

## 1 Introduction

Canavan disease (CD) is a rare lethal leukodystrophy caused by genetic mutations in the aspartoacylase (*ASPA*) gene.^[^ ^1^ ^]^ The ASPA protein is a metabolic enzyme that is mainly expressed in oligodendrocytes.^[^ ^2^ ^]^ The ASPA substrate *N*‐acetyl‐aspartate (NAA) is a small molecule (an amino acid) with a molecular weight of only 174.2 Da and is mainly produced in neurons. It is one of the most abundant and concentrated metabolites in the brain. The NAA generated by neurons can be transferred to oligodendrocytes and hydrolyzed by the ASPA enzyme in oligodendrocytes to maintain the NAA concentration in a physiologically relevant level in the brain.^[^ ^1^, ^3^ ^]^ The deficiency of the ASPA enzyme causes NAA accumulation in the brain and the cerebrospinal fluid (CSF).^[^ ^4^ ^]^ When the brain NAA reaches a high level, it is excreted to the blood and urine, leading to elevated NAA level in the blood and urine in CD patients.^[^ ^5^ ^]^ It has been proposed that a high concentration of NAA can be toxic to myelin forming oligodendrocytes, resulting in hypomyelination and spongy degeneration (vacuolation) in the brain.^[^ ^3^ ^]^ Furthermore, several clinical symptoms including impaired motor function, mental retardation, and early death are observed in CD patients.^[^ ^6^ ^]^ CD patients with the infantile‐onset form are most prevalent and often die in the first decade of their lives.^[^ ^5^ ^]^

There is currently no approved therapy for CD. Patient induced pluripotent stem cell (iPSC)‐derived autologous cellular products have great potential for cell therapy development because of the unlimited cell source that can be obtained from iPSCs and the reduced potential of immune rejection.^[^ ^7^ ^]^ In our previous study, a functional *ASPA* gene was introduced into patient iPSC‐derived neural progenitor cells (NPCs) or oligodendrocyte progenitor cells (OPCs) via lentiviral transduction or transcription activator‐like effector nuclease (TALEN)‐mediated genetic engineering to generate ASPA NPC or ASPA OPC. The modified cells were transplanted into the brains of CD (Nur7) mice and resulted in sustained rescue of multiple disease phenotypes.^[^ ^8^ ^]^ However, manufacturing cellular products tailored for each patient can be time‐consuming, which in turn can limit wide clinical applications. An allogeneic cellular product that can be stored after extensive releasing tests offer an “off‐the‐shelf” product that is readily available, thus representing an appealing option for cell therapy development, especially for time‐sensitive diseases and common disorders.

One major concern of allogenic cell therapy is human leukocyte antigen (HLA) mismatch‐induced immune rejection.^[^ ^7b^ ^]^ “Off‐the‐shelf” hypoimmunogenic cells have been created to overcome this limitation.^[^ ^9^ ^]^ HLA class I (HLA‐I) and HLA class II (HLA‐II) have been depleted to evade the attack from CD8^+^ cytotoxic T cells and CD4^+^ T helper cells.^[^ ^7^, ^10^ ^]^ To prevent cell lysis by natural killer (NK) cells after knocking out HLA‐I, retention or overexpression of part of the HLA regimen, such as HLA‐C, HLA‐E or HLA‐G, or expression of immune‐suppressive molecules, have been designed to protect the engineered cells.^[^ ^10^, ^11^ ^]^ Because the immune checkpoint proteins such as programmed death‐ligand 1 (PD‐L1), programmed cell death protein 1 (PD‐1), cluster of differentiation 47 (CD47), and cytotoxic T‐lymphocyte‐associated antigen 4 (CTLA‐4) can regulate NK activity in normal and cancer cells,^[^ ^12^ ^]^ expression of CD47 and PD‐L1 in HLA‐I knockout cells has been employed to suppress the NK response.^[^ ^10b,c^ ^]^ These approaches have been used to generate hypoimmunogenic islet cells, chimeric antigen receptor (CAR)‐T cells, and other cell types from human pluripotent stem cell (PSC)s that can escape the recognition from allogeneic immune cells.^[^ ^9^, ^10^, ^13^ ^]^ However, there is no report on using hypoimmunogenic cells as universal donors for cell therapy development for diseases of the central nervous system yet.

Stem cell therapy with OPCs, the precursor cells of oligodendrocytes, is a promising choice for demyelinating disorders.^[^ ^14^ ^]^ OPCs can be efficiently derived from human embryonic stem cells (ESCs) and human iPSCs.^[^ ^8^, ^15^ ^]^ After intracerebral engraftment, OPCs can migrate extensively, mature into oligodendrocytes, and myelinate demyelinated loci throughout the mouse brain.^[^ ^8^, ^15^ ^]^ Engrafted OPCs can enhance animal survival with progressive resolution of neurological defects in myelin‐deficient shiverer mice,^[^ ^15^, ^16^ ^]^ and restore myelination and behavioral deficits of irradiated rats that exhibit OPC depletion and demyelination after brain radiation.^[^ ^15d^ ^]^ ASPA is naturally expressed in oligodendrocytes which are differentiated from OPCs in the brain.^[^ ^2^ ^]^ However, previous therapies including viral therapies^[^ ^3^, ^17^ ^]^ and ex‐vivo stem cell therapies^[^ ^8^, ^15^ ^]^ need to introduce an exogenous gene into the genome, which may raise potential safety concern. OPCs derived from health donor (HD) iPSCs carrying the wildtype (WT) *ASPA* gene can be an ideal cell therapy candidate to rescue pathological phenotypes in CD, especially when combined with the hypoimmunogenicity.

In this study, hypoimmunogenic iPSCs were generated from HD iPSCs that carry the endogenous WT *ASPA* gene through CRISPR/Cas9‐based editing of the HLA molecules and differentiated into OPCs. After being transplanted into the brains of an immunodeficient CD (Nur7) mouse model, the survival and distribution of the hypoimmunogenic OPCs were examined, and the preclinical efficacy was evaluated. Furthermore, the immunogenicity of the hypoimmunogenic OPCs was assessed both in vitro and in vivo.

## 2 Results

### 2.1 Hypoimmunogenic OPCs Lack Surface Expression of HLA‐I and HLA‐II

**Figure 1:**
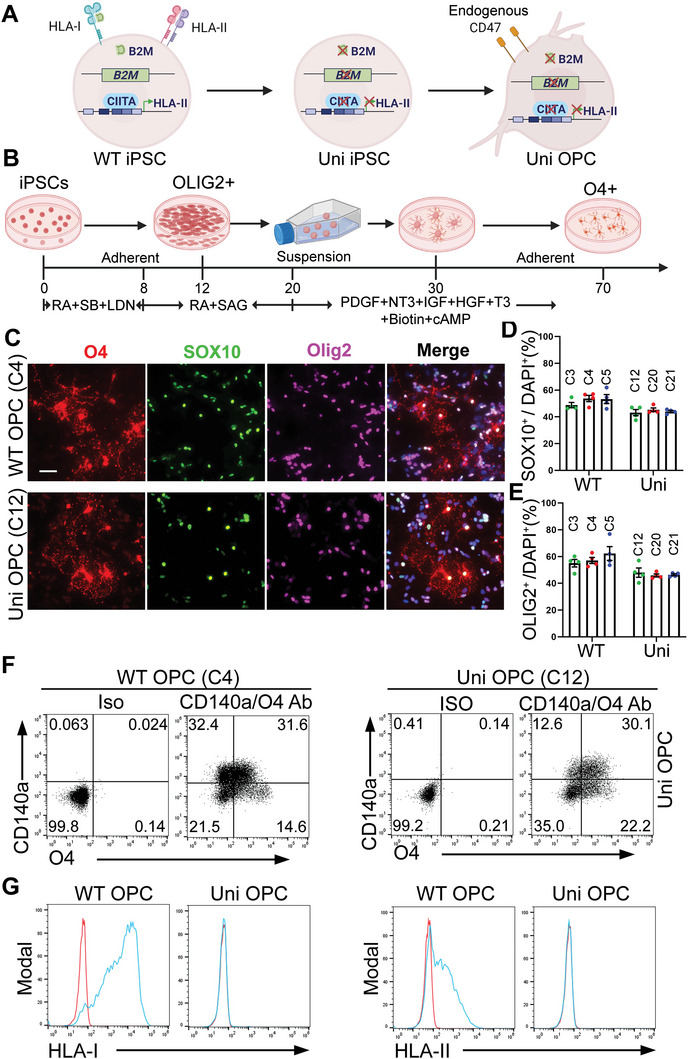
Generation and characterization of hypoimmunogenic universal (uni) OPCs. A) A schematic for the strategy of gene editing to generate hypoimmunogenic iPSCs and OPCs. B) A schematic protocol for differentiating human iPSCs into OPCs. C) Immunostaining of the WT and uni OPCs for the oligodendroglial lineage markers O4, SOX10, and OLIG2. Scale bar: 50 µm. D,E) The percentage of SOX10^+^ cells and OLIG2^+^ cells were quantified, respectively. *n* = 4 images for each clone, three clones for each cell line. F) Flow cytometry analysis of the WT (C4) or uni (C12) OPCs using the OPC markers CD140a and O4. The isotype IgG was included as the negative control. G) The lack of HLA‐I and HLA‐II expression on uni OPCs. Flow cytometry analysis of the WT (C4) or uni (C12) OPCs stained with the HLA‐I or HLA‐II antibodies (blue line) after IFN‐*γ* treatment. The isotype IgG was included as the negative control (red line).

Because immune rejection is one of the main challenges for the development of allogeneic cell therapies, we aimed to generate hypoimmunogenic OPCs as a cell therapy product for CD to reduce the risk of immune rejection. Because CD is caused by genetic mutation of the *ASPA* gene, we proposed to generate OPCs carrying the wild type (WT) *ASPA* gene so that they can express the functional ASPA enzyme. To generate hypoimmunogenic iPSCs with the endogenous WT *ASPA* gene, we used human iPSCs derived from a health donor (HD iPSC) that carries the WT *ASPA* gene as the starting cells (**Figure** **1**A). Because mismatched HLA‐I recognized by CD8^+^ cytotoxic T cells and mismatched HLA‐II recognized by CD4^+^ helper T cells are the main cause of immune rejection, we impaired the expression of HLA‐I and HLA‐II by disrupting the *β*‐2‐microglobulin (*B2M)* gene that encodes a structural component of HLA‐I and the class II major histocompatibility complex transactivator (*CIITA)* gene that encodes the master regulator of HLA‐II genes (Figure 1A).^[^ ^9^, ^10^ ^]^ Specifically, we simultaneously knocked out the *B2M* and the *CIITA* genes in HD iPSCs by transfecting the Cas9 protein and two sgRNAs that target the *B2M* and the *CIITA* gene, respectively. iPSC clones arisen from single cells were picked and sequenced. A total of 36 clones were picked, from which 26 clones were confirmed to have mutation in both genes by Sanger sequencing. Because of the hypoimmunogenic potential of the *B2M^−/−^CIITA^−/−^* iPSCs, they may be used as universal donors. Therefore, we termed these iPSCs as “universal iPSCs” for short (Figure 1A). The parental HD iPSCs that carry the WT *B2M* and *CIITA* genes were termed “WT iPSCs”. Because the universal iPSCs were generated from the WT iPSCs, the WT iPSCs are considered an isogenic control for the universal iPSCs.

Four clones with frameshift mutations in both the *B2M* gene and the *CIITA* gene were selected. The expression of HLA‐I was examined by flow cytometry on gene‐edited‐iPSCs treated with IFN‐*γ*. The surface expression of HLA‐I was lost largely on all four clones of universal iPSCs (Figure S1A,B, Supporting Information). The expression of HLA‐II was determined after differentiation of iPSCs into OPCs (see below) because of the low expression level of HLA‐II ion iPSCs.

We differentiated both the WT and the universal iPSCs into OPCs following a published protocol (Figure 1B).^[^ ^8^, ^15^ ^]^ Clone‐3, 4 and 5 of WT iPSCs and clone‐12, 20 and 21 of universal iPSCs were used for differentiation. After 70 days of differentiation, oligodendrocyte marker O4‐positive OPCs were detected by live staining (Figure S1C, Supporting Information). Both the WT and the universal OPCs expressed oligodendrocyte transcription factor 2 (OLIG2) and SRY‐box transcription factor 10 (SOX10) besides O4 as revealed by immunostaining (Figure 1C; Figure S1D, Supporting Information). The WT and universal iPSCs, including multiple clones from each, showed comparable differentiation trend (Figure 1C–E; Figure S1C,D, Supporting Information). Clone‐4 of WT iPSCs and clone‐12 of universal OPCs were randomly chosen for the following characterization and functional study. Staining the WT and the universal OPC populations with markers for different neural lineages, including SOX10 for oligodendroglial cells, SRY‐box transcription factor 9 (SOX9) for astrocytes, microtubule associated protein 2 (MAP2) for neurons, and paired box 6 (PAX6) for neural progenitor cells (NPCs), revealed that they were mainly composed of SOX10^+^ oligodendroglial cells (45.90 ± 1.58% for WT OPCs and 47.04 ± 3.46% for universal OPCs), with a small population of SOX9^+^ astrocytes (10.08 ± 0.66% for WT OPCs and 13.25 ± 1.70% for universal OPCs), and barely any MAP2^+^ neurons and PAX6^+^ NPCs (Figure S2, Supporting Information). Flow cytometry analysis revealed platelet derived growth factor receptor alpha (CD140a) single positive cells, O4 single positive cells, and CD140a and O4 double positives cells in both the WT and universal OPC populations (Figure 1F), consistent with the previous observation that OPCs can be differentiated in multiple overlapping waves^[^ ^15d^ ^]^ and the differentiated cells include OPCs at different stages.^[^ ^18^ ^]^ These results indicate that knockout of the *B2M* and *CIITA* genes does not affect OPC differentiation. The surface expression of the HLA‐I and HLA‐II on universal OPCs was determined by flow cytometry. No surface expression of HLA‐I and HLA‐II was detected on universal OPCs treated with IFN‐*γ*, while the expression was readily detectable on WT OPCs (Figure 1G).

### 2.2 Universal OPCs Exhibit Widespread Distribution in Transplanted CD (Nur7) Mouse Brains

**Figure 2:**
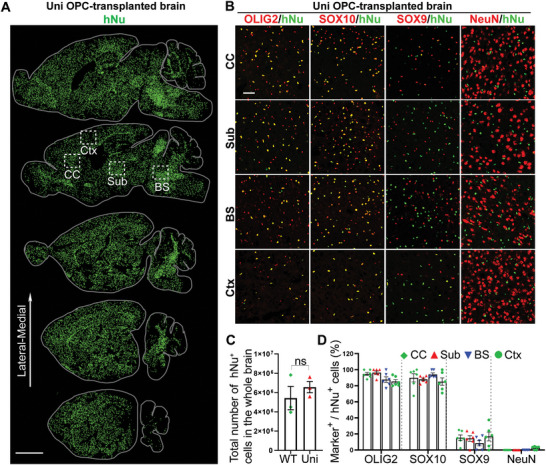
The uni OPCs exhibit widespread distribution after being transplanted into CD (Nur7) mouse brains. A) The uni OPCs migrated and spread widely in the brain six months after transplantation. The serial sagittal dot maps of the human nuclear antigen (hNu) staining are shown. Each series was begun from the lateral to the midline and continued at 900 µm intervals. Scale bar: 2000 µm. B) The uni OPCs gave rise to mostly oligodendroglial lineage cells, a small population of astrocytes and few neurons in the transplanted mouse brains. Six months after transplantation, the uni OPC‐transplanted brains were immunostained for hNu and the oligodendroglial lineage markers OLIG2 and SOX10, the astrocyte marker SOX9 and the neuronal marker NeuN. Images from the corpus callosum (CC), the subcortical white matter (Sub), the brain stem (BS) and the cortex (Ctx) were shown. Scale bar: 50 µm. C) The total hNu^+^ human cells in whole brain from WT and uni OPC‐transplanted mice. *n* = 3 mice for WT and uni OPC‐transplanted mice. ns, not significant by Student's *t*‐test (two‐tailed). D) The percentage of the neural lineage marker^+^ cells in the transplanted hNu^+^ human cells. The hNu^+^ neural lineage marker^+^ cells in four regions of the brain, including the corpus callosum (CC), the subcortical white matter (Sub), the brain stem (BS) and the cortex (Ctx) were quantified. *n* = 6 fields from each region from 4 mice, with 2 images from each region in each mouse brain.

The universal OPCs were transplanted into an immunodeficient CD mouse model for evaluating their effects on rescuing disease phenotypes. The Aspa^nur7/nur7^/Rag2^−/−^ mice, termed “CD (Nur7) mice”, exhibit characteristic CD disease phenotypes and have been used as an authentic CD mouse model for multiple studies,^[^ ^8^, ^15^, ^19^ ^]^ therefore, were chosen for this study. Both the universal and the WT OPCs were transplanted into the brains of postnatal day 1 to 4 (P1‐4) CD (Nur7) mice bilaterally at the corpus callosum, the subcortical white matter, and the brain stem as we described before.^[^ ^8^, ^15^ ^]^ Six months after transplantation, brains of the transplanted mice were harvested and analyzed. The survival and distribution of the OPCs in brains of the transplanted mice were determined by immunohistochemical staining for human nuclear antigen (hNu). The hNu signal was clearly detected and widely distributed all over the brain (**Figure** **2**A; Figure S3A, Supporting Information), indicating that both the WT and the universal OPCs can survive and migrate extensively in the transplanted CD (Nur7) mouse brains. Counting hNu^+^ cells throughout the brain revealed that both the WT and the universal OPCs were expanded after transplantation. The cell number was increased from 9×10^5^ cells at the time of transplantation to 5.42×10^6^ ± 1.21×10^6^ for WT OPCs and 6.56×10^6^ ± 0.58×10^6^ for WT OPCs 6 months after transplantation. There was no significant difference in the number of engrafted cells between WT and universal OPCs (Figure 2C). The fate of the transplanted OPCs was evaluated by co‐staining of the transplanted brains for hNu and different neural lineage markers. The result showed that most donor cells were oligodendroglial lineage cells (hNu^+^OLIG2^+^ and hNu^+^SOX10^+^), a small population of cells were astrocytes (hNu^+^SOX9^+^), and only very few donor cells were neurons (hNu^+^NeuN^+^) (Figure 2B,D; Figure S3B,C, Supporting Information). There was no obvious difference in the fate of the transplanted cells in different regions of the brain, including the corpus callosum, the subcortical, the brain stem white matter region, as well as the cortex (Figure 2D; Figure S3C, Supporting Information). The survival and the fate of the WT and the universal OPCs are comparable in the transplanted brains (Figure 2B–D; Figure S3B,C, Supporting Information).

### 2.3 The Grafted Universal OPCs Express the ASPA Enzyme and Reduce the NAA Level in CD (Nur7) Mouse Brains

**Figure 3:**
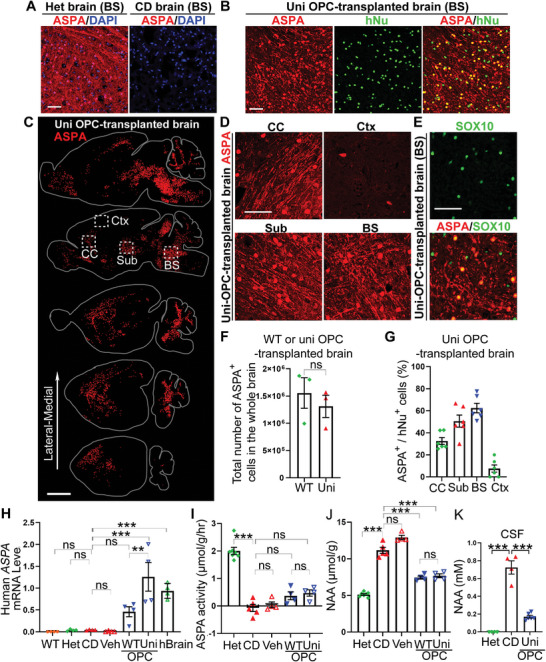
Widely expressed ASPA and reduced NAA level in the WT and uni OPC‐transplanted CD (Nur7) mouse brains. A) Lack of the ASPA protein expression in CD (Nur7) mice due to the nonsense mutation in the *ASPA* gene. Immunostaining showed that the heterozygous (Het) mouse brains showed widespread ASPA expression, whereas no ASPA expression could be detected in the CD (nur7) mouse brains. Scale bar: 50 µm. B) Immunostaining for ASPA showed that the uni OPC‐transplanted CD (Nur7) mouse brains expressed ASPA. The ASPA expression in the cell body was colocalized with hNu. Scale bar: 50 µm. C) A serial sagittal dot maps showing widespread ASPA expression in the white matter track of the brain based on the immunostaining signal of ASPA in the cell body. Each series was begun from the lateral to the midline and continued at 900 µm intervals. Scale bar: 2000 µm. D) The ASPA staining images from the boxed regions in panel (D) are shown. In addition to cell body expression, ASPA was also expressed in the processes of oligodendrocytes. Shown are images from three regions, including the corpus callosum (CC), the subcortical white matter (Sub), the brain stem (BS), and the cortex (Ctx). Scale bar: 50 µm. E) Co‐expression of ASPA with the oligodendroglial lineage marker SOX10 in the uni OPC‐transplanted CD (Nur7) mouse brains. Scale bar: 50 µm. F) The total ASPA^+^ cells in the whole brain from WT and uni OPC‐transplanted mice. *n* = 3 mice for WT and uni OPC‐transplanted mice. G) The percentage of ASPA^+^ oligodendrocyte cells in the uni OPC (hNu^+^) ‐transplanted brains. About half of the human cells matured into oligodendrocytes and expressed the ASPA enzyme in the white matter regions, but low percentage in the cortex. *n* = 8 fields from 3 mice, with 2 images from each region in each mouse brain. H) Expression of the human *ASPA* mRNA in the WT and uni OPC‐transplanted mouse brains. The human cortex brain tissues were included as a positive control. Each dot represents the result from an individual mouse or human sample. *n* = 4 mice or human brain samples. I) Elevated ASPA activity and J) reduced NAA level in the WT and uni OPC‐transplanted CD (Nur7) mouse brains six months after transplantation. The NAA level was measured using NMR. The ASPA activity was measured by NMR and expressed as reduced NAA level per gram (g) of brain tissue within an hour (hr) (µmol/g/hr). Each dot represents the result from an individual mouse. *n* = 7 mice for the Het, 5 for the CD (Nur7) mice, 4 for the vehicle (Veh) control‐treated mice, 4 for the WT and uni OPC‐transplanted mice, respectively, for panels (I) and (J). K) Reduced NAA level in the cerebrospinal fluid (CSF) in the uni OPC‐transplanted CD (Nur7) mouse six months after transplantation. *n* = 4 mice for the Het and the CD (Nur7) mice, 6 for uni OPC‐transplanted mice, respectively, for panel (J). Error bars are SE of the mean. ns, not significant, **p < 0.01, ***p < 0.001 by Student's *t*‐test (two‐tailed) for panel (F), one‐way ANOVA followed by Dunnett's multiple comparisons test for panels (H) and (K), or by Tukey's multiple comparisons test for panels I and J.

The deficiency of ASPA enzyme is the underlying cause of CD. The CD (Nur7) mouse model carries a nonsense mutation, Q193X, in the *ASPA* gene, resulting in a non‐functional, truncated ASPA protein that is not detectable by the ASPA antibody used in the previous study^[^ ^20a^ ^]^ and this study (**Figure** **3**A). We checked whether universal OPCs could restore ASPA expression after being transplanted into the brains of CD (Nur7) mice. Immunostaining revealed widespread ASPA expression in the heterozygous (Het) mouse brains, but no ASPA expression in the CD (Nur7) mouse brains (Figure 3A). Robust ASPA expression in both the cell body and the processes was observed in the universal OPC‐transplanted CD (Nur7) mouse brains and the ASPA signal in the cell body was colocalized with the hNu signal, similar to that in the WT OPC‐transplanted brains (Figure 3B; Figure S4A, Supporting Information). Double staining of the transplanted brains for ASPA and the oligodendroglial marker SOX10 (Figure 3E; Figure S4A, Supporting Information) or the mature oligodendrocyte marker CC1 (Figure S4B, Supporting Information) revealed that ASPA was expressed in mature oligodendrocytes. Indeed, ASPA has been used as a mature oligodendrocyte marker in previous studies.^[^ ^20^ ^]^ Widespread distribution of the ASPA‐positive oligodendrocytes was observed in both the WT and the universal OPC‐transplanted CD (Nur7) mouse brains (Figure 3C,D; Figure S4C,D, Supporting Information). Unlike the whole brain distribution of the hNu‐positive cells, the ASPA‐positive oligodendrocytes were mostly distributed in the white matter tracks, among which the subcortical white matter and the brain stem are also the main regions with severe vacuolation (Figure 3C,D; Figures S4C,D and S5, Supporting Information). The distribution of the ASPA‐positive cells is consistent with the developmental pattern of myelination in the brain.^[^ ^21^ ^]^ There was no significant difference in the total number of ASPA‐positive oligodendrocytes in the WT OPC‐transplanted and the universal OPC‐transplanted brains (Figure 3F). About half of the hNu‐positive cells matured into ASPA^+^ oligodendrocytes (48.53 ± 3.82% hNu^+^ASPA^+^ cells for universal OPCs, and 53.13 ± 3.30% hNu^+^ASPA^+^ cells for WT OPCs, Figure 3G and Figure S4E, Supporting Information) in the white matter tracks. The expression of ASPA was detected in both the cell body and the processes (Figure 3B,D,E; Figure S4D, Supporting Information). The reason that we were able to detect ASPA expression in the processes in the transplanted brains is presumably because of the lower density of the ASPA‐positive oligodendrocytes in the transplanted brains compared to that in the Het brains. These results together indicate that the milieu of the CD (Nur7) mouse brains is not harmful to the transplanted OPCs, allowing the survival and maturation of these OPCs into oligodendrocytes for remyelination.

The expression of human *ASPA* in the WT OPC or universal OPC‐transplanted CD (Nur7) mouse brains was confirmed by quantitative RT‐PCR (qRT‐PCR) using the human‐specific ASPA primers. Human *ASPA* mRNA was detected in the WT OPC or universal OPC‐transplanted CD (Nur7) mouse brains, but not in the brains of control mice without OPC transplantation (Figure 3H). The expression of ASPA in the human cortex was included as a positive control.

The detection of ASPA expression led us to determine the ASPA enzymatic activity in the OPC‐transplanted CD (Nur7) mouse brains. Both the un‐treated CD (Nur7) mice and the vehicle‐treated CD (Nur7) mice exhibited deficient ASPA activity in the brains (Figure 3I). Both the WT OPCs and the universal OPCs increased the ASPA enzymatic activity in the transplanted CD (Nur7) mouse brains, compared to that in the un‐treated or vehicle‐treated CD (Nur7) mouse brains, although the activity in the transplanted brains is lower than that in the Het mouse brains (Figure 3I), presumably because the ASPA^+^ cell density in the transplanted brains is lower than that in the Het mouse brains (Figure 3A–C).

Because ASPA is the enzyme that catabolizes NAA, we next tested if the NAA level in the transplanted brains was altered. Both the un‐treated and the vehicle‐treated CD (Nur7) mice exhibited high level of NAA in the brains, compared to the Het mice (Figure 3I,J). Of interest to us, the mildly elevated ASPA activity in the WT OPC‐transplanted or the universal OPC‐transplanted CD (Nur7) mouse brains was able to reduce NAA level substantially, compared to that in the un‐treated and the vehicle‐treated CD (Nur7) mouse brains (Figure 3I,J), presumably because of the long‐term effect from the sustained ASPA activity. Moreover, transplantation with the universal OPCs reduced the NAA level in the cerebrospinal fluid (CSF) of the CD (Nur7) mice substantially (Figure 3K). These results together indicate that the universal OPCs were able to reconstitute the ASPA enzymatic activity and reduce NAA level in the transplanted CD (Nur7) mouse brains, in a manner that is similar to the WT OPCs.

### 2.4 The Grafted Universal OPCs Allow Remyelination in Transplanted CD (Nur7) Mouse Brains

**Figure 4:**
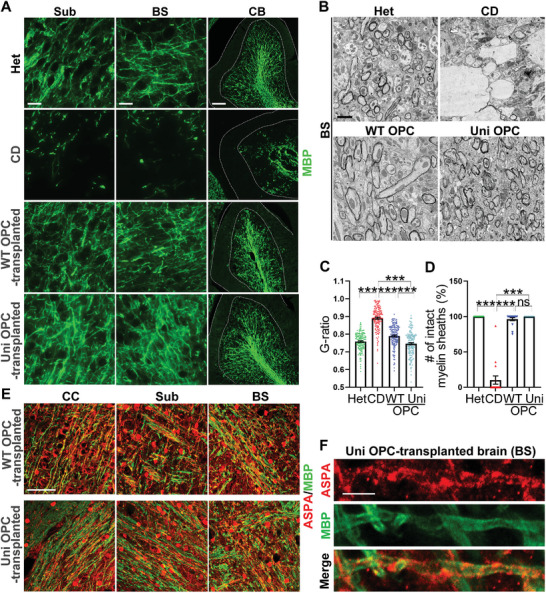
Improved myelination in the WT and uni OPC‐transplanted CD (Nur7) mouse brains. A) Improved myelination in the WT and uni OPC‐transplanted CD (Nur7) mouse brains was shown by MBP staining. Shown are images from three regions, including the corpus callosum (CC), the subcortical white matter (Sub), and the cerebellum (CB). Scale bar: 25 µm for Sub and BS, 100 µm for CB in panel (B). B) Improved myelination as shown by electron microscopy and revealed by the increased number of intact myelin sheaths and the enhanced thickness of myelin sheaths in brains of the transplanted mice, compared to the control CD (Nur7) mice. Images of the subcortical white matter are shown. Scale bar: 2 µm. C,D) Quantification showing enhanced thickness of myelin sheaths as revealed by reduced G‐ratio (C) and increased number of intact myelin sheaths (D) in brains of the WT and uni OPC‐transplanted CD (Nur7) mice, compared to that in the control CD (Nur7) mice. *n* = 50 myelin sheaths from each region. Three regions, including the subcortical white matter, the brain stem and the cerebellum white matter from one mouse brain of each group were analyzed. E) Co‐localization of MBP and ASPA in different regions of the WT and uni OPC‐transplanted brains. Scale bar, 50 µm. F) Enlarged images showing the co‐localization of ASPA and MBP on the processes of oligodendrocytes. Scale bar: 5 µm. Error bars are SE of the mean. ns, not significant, ***p < 0.001 by one‐way ANOVA followed by Tukey's multiple comparisons test.

Because CD is a demyelinating disease, we next asked if the universal OPCs can rescue the myelination defects in the transplanted mouse brains. To check myelination in the brain, we did MBP immunostaining, osmium tetroxide staining, and electron microscopy (EM) analysis to exam myelin sheaths from the overall phenotype to the ultrastructure. The CD (Nur7) mouse brains exhibited substantially less myelin sheaths in the vacuolated regions and disrupted myelin sheaths even in the regions without vacuolation based on the MBP immunostaining (**Figure** **4**A; Figure S5A, Supporting Information). Transplantation with the universal OPCs increased myelination substantially, similar to transplantation with the WT OPCs. Well‐organized myelin sheaths were detected in multiple regions of the transplanted brains (Figure 4A; Figure S5A, Supporting Information). Compared to the control CD (Nur7) mice, both the WT and the universal OPCs had markedly increased the MBP labeling intensity and the MBP^+^ area (Figure S5B,C, Supporting Information). Remyelination and restored myelin sheath fiber tracks were also observed by Osmium Tetroxide staining, a widely used stain for lipids in membranous structures (Figure S6A,B, Supporting Information), consistent with the result from MBP staining (Figure 4A; Figure S5A, Supporting Information). Furthermore, the ultrastructure of the myelin sheaths was detected by EM imaging. Compared to the vacuolated myelin sheaths found in control CD brains, healthy myelin sheaths with well‐organized wrapping were found in the universal OPC or the WT OPC‐transplanted CD mouse brains (Figure 4B). While the percentage of well‐wrapped myelin sheaths was much lower in CD (Nur7) mouse brains compared to that in the heterozygous mouse brains, transplantation with either the universal OPCs or the WT OPCs led to a substantially increased percentage of normal myelin sheaths in the CD (Nur7) mouse brains (Figure 4B,D). The universal OPC or WT OPC‐transplanted CD mouse brains also contained myelin sheaths with much reduced G ratio, the ratio of the inner diameter to the outer diameter of myelin sheaths, compared to that in the control CD brains (Figure 4C), indicating that the transplanted brains contain thicker myelin sheaths. Together, these results demonstrate that transplantation with the universal OPCs were able to rescue myelination deficits in CD (Nur7) mouse brains, similar to transplantation with the WT OPCs.

The expression of ASPA in the myelin sheaths of the transplanted brains suggests that the transplanted universal or WT OPCs may contribute to remyelination after maturing into oligodendrocytes (Figure 3D; Figure S4D, Supporting Information). Indeed, co‐expression of ASPA with MBP was detected on myelin sheaths in multiple regions of the transplanted brains (Figure 4E). Higher magnification images revealed ASPA expression in tube‐like structures and colocalization of ASPA and MBP on myelin sheaths (Figure 4F; Figure S4F, Supporting Information). These results together indicate that both the universal OPCs and the WT OPCs can give rise to mature oligodendrocytes and contribute to remyelination along with endogenous mouse oligodendrocytes. Therefore, the universal OPCs can provide not only functional ASPA enzyme to reduce NAA level but also become oligodendrocytes for remyelination, similar to the WT OPCs.

### 2.5 The Grafted Universal OPCs can Rescue Spongy Degeneration in CD (Nur7) Mouse Brains

**Figure 5:**
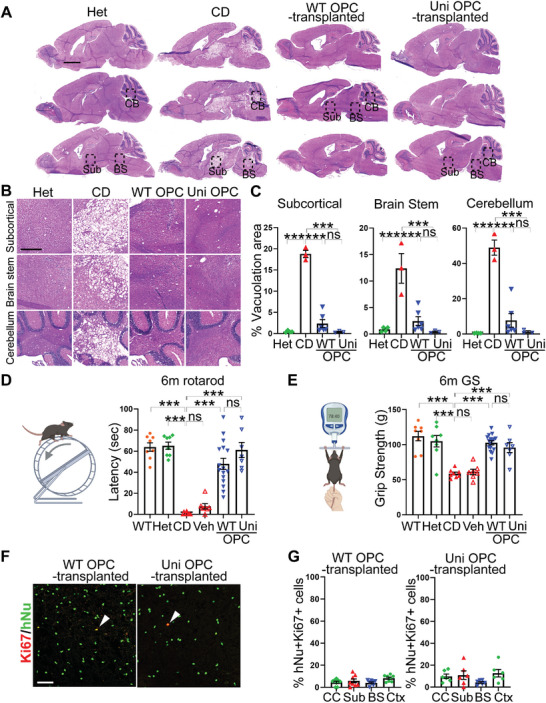
Reduced vacuolation and improved motor function in the WT and uni OPC‐transplanted CD (Nur7) mice. A) Reduced vacuolation in the WT and uni OPC‐transplanted CD (Nur7) mouse brains six months after transplantation as revealed by H&E staining. Three sagittal whole brain sections from one mouse are shown in each group. The heterozygous (Het) mice were included as the positive control and the homozygous CD (Nur7) mice as the negative control. Scale bar: 2000 µm. B) Enlarged H&E images of the subcortical white matter, the brain stem, and the cerebellum from the boxed regions in panel (A) are shown. Scale bar: 500 µm. C) Quantification of the vacuolation area in the subcortical, the brain stem, and the cerebellum white matter. *n* = 4 mice for Het, *n* = 3 mice for CD, *n* = 6 mice for the WT OPC‐transplanted group, and *n* = 3 mice for the uni OPC‐transplanted group. D,E) Improved motor function in WT and uni OPC‐transplanted CD (Nur7) mice as revealed by the rotarod and grip strength tests. Each dot represents the result from an individual mouse. *n* = 8 mice for the WT, Het, and CD (Nur7) mice, *n* = 7 mice for vehicle control‐treated mice, *n* = 16 for the WT OPC‐transplanted mice, and *n* = 7 for the uni OPC‐transplanted mice. F,G) The WT and uni OPCs showed low mitotic index in the transplanted mouse brains as revealed by hNu and Ki67 co‐staining and quantification. Scale bar: 50 µm. Error bars are SE of the mean. ns, not significant, *p < 0.05, **p < 0.01, and ***p < 0.001 by one‐way ANOVA followed by Tukey's multiple comparisons test.

Spongy degeneration, also described as vacuolation, is a prominent pathological feature observed in CD patients.^[^ ^5^, ^22^ ^]^ To determine if the universal OPCs were able to recuse the spongy degeneration in CD (Nur7) mice, serial sagittal sections from the control or the universal OPC‐transplanted mouse brains were evaluated for vacuolation by H&E staining. As in patients, extensive vacuolation was observed in multiple regions of the CD (Nur7) mouse brains, including the subcortical white matter, the brain stem, and the cerebellum (**Figure** **5**A–C), consistent with the observation in previous studies.^[^ ^8^, ^15^, ^19^ ^]^ In contrast, the universal OPC‐transplanted brains exhibited substantially reduced vacuolation and intact brain structure, similar to that in the WT OPC‐transplanted CD brains or the Het brains (Figure 5A–C). Importantly, the cerebellum region was also largely rescued, even though it is far away with the injection site.^[^ ^8^ ^]^ Presumably, the extensive migration of the transplanted OPCs allowed the cells to reach distant regions to enable rescue via either reducing the NAA level and/or cellular effects. These results indicate that the universal OPCs allow widespread rescue of the spongy degeneration in the CD (Nur7) mouse brains, similar to the WT OPCs.

### 2.6 The Grafted Universal OPCs Rescue Gross Motor Function in Transplanted CD (Nur7) Mice

CD patients often fail to develop motor control (Canavan disease in NORD, Reuben Matalon, https://rarediseases.org/rare‐diseases/canavan‐disease). CD animal models also exhibit these functional deficits. To evaluate the effect of the universal OPCs on motor performance in transplanted CD (Nur7) mice, we tested the universal OPC‐transplanted CD (Nur7) mice in two motor skill paradigms: accelerating rotarod test (Figure 5D) for testing motor coordination and balance and grip strength test (Figure 5E) for evaluating the forepaw strength as an indication of neuromuscular function.^[^ ^23^, ^24^ ^]^ The un‐treated or vehicle‐treated CD (Nur7) mice were included as the negative control, while the WT OPC‐transplanted mice were included as the positive control. In contrast to the un‐treated or vehicle‐treated control CD (Nur7) mice that exhibited impaired rotarod performance and grip strength compared to the WT and the Het mice (Figure 5D,E), transplantation with the universal OPCs improved the rotarod performance and enhanced the grip strength substantially in CD (Nur7) mice, similar to transplantation with the WT OPCs (Figure 5D,E). These results demonstrate that the universal OPCs can improve motor functions in CD mice. Moreover, only a small fraction of the transplanted cells remained mitotically competent 6 months after transplantation, as revealed by hNu and Ki67 double staining (Figure 5F,G). Based on the in vitro staining of OPCs for cell type‐specific markers and Ki67 before transplantation (Figure S2, Supporting Information), the Ki67+ cells in the transplanted CD mice are likely OPCs or astrocytes. No tumor was found in the transplanted brain sections. These results together indicate that the universal OPCs are highly efficacious in rescuing the pathological features of CD in a preclinical mouse model and have a preliminary safety profile, therefore representing a promising cell therapy candidate for CD.

### 2.7 Universal OPCs Exhibit Low Immunogenicity In Vitro

**Figure 6:**
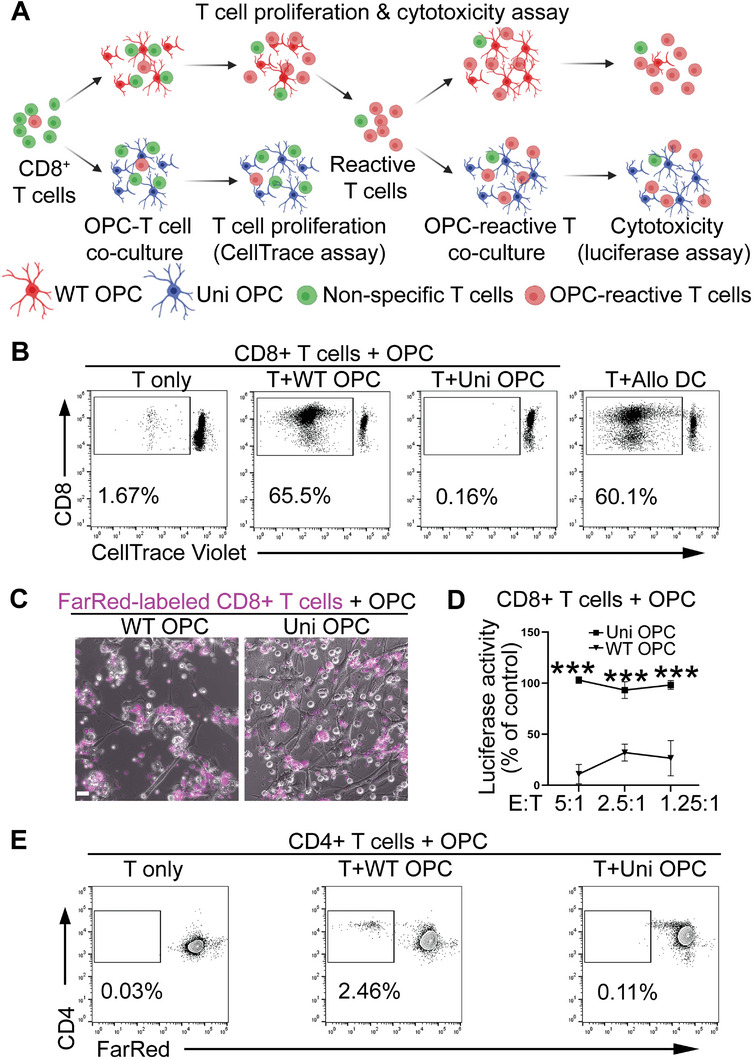
The hypoimmunogenic universal (uni) OPCs exhibit low immunogenicity. A) A schematic for evaluating the immunogenicity of cytotoxic CD8^+^ T cells. The WT or uni OPCs were co‐cultured with CD8^+^ T cells isolated from allogenic PBMC and the proliferation of CD8^+^ T cells was tracked by the CellTrace dye. The reactive T cells were incubated with the WT or uni OPCs to assess CD8^+^ T cell cytotoxicity by a luciferase assay. B) The CD8^+^ T cells were expanded in response to the WT but not the uni OPCs. The CellTrace proliferation assay was used to detect the proliferation of CD8^+^ T cells in response to the WT or uni OPCs. The allogeneic dendritic cells (DC) were included as the positive control, while the CD8^+^ T cells only as the negative control. The CD8^+^ T cells were labeled with the CellTrace dye and the proliferated CD8^+^ T cells with low intensity of the CellTrace dye were gated. C,D) The WT but not uni OPCs were lysed by CD8+ T cells. The WT or uni OPCs carrying a luciferase reporter were co‐cultured with reactive CD8^+^ T cells labeled by the CellTrace‐FarRed dye for 48 h. Images are shown in panel (C). Luciferase activity are shown in panel (D). Scale bar: 100 µm. E) The universal OPCs exhibited low immunogenicity for CD4^+^ T cells. The CellTrace proliferation assay was performed to detect proliferative CD4^+^ T cells in response to on the WT or uni OPCs. CD4^+^ T cells were labeled with CellTrace‐FarRed and proliferated CD4^+^ T cells with low intensity of CellTrace dye were gated. CD8^+^ T cells and CD4^+^ T cells isolated from Donor 3 were used for Figure 2 ***p < 0.001 by two‐way ANOVA followed by Benferroni's multiple comparisons test for panel (D).

**Figure 7:**
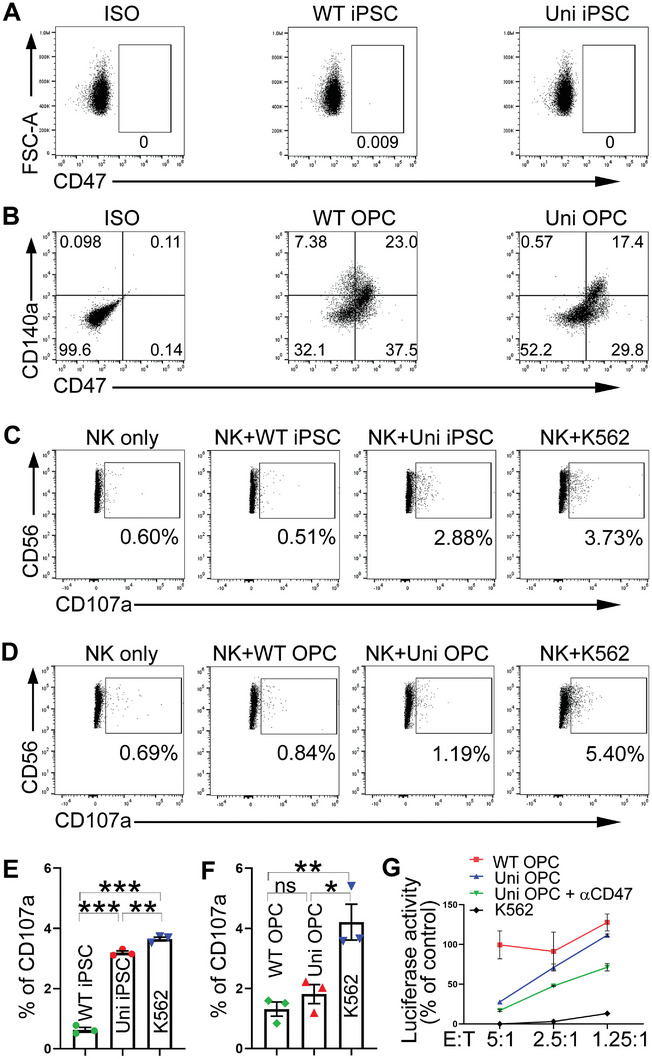
The uni OPCs partly evade NK response. A) Both the WT and the uni iPSCs didn't express CD47 based on the Flow cytometry analysis. B) Both the WT and the uni OPCs expressed CD47. Flow cytometry analysis of the WT or the uni OPCs stained with antibodies for CD140a and CD47. C–F) The uni OPCs, not uni iPSCs were able to evade the NK response. The WT or uni iPSCs and OPCs were co‐cultured with NK cells isolated from allogenic PBMC. The degranulation assay by flow cytometry analysis of CD107a expression to assess NK cell activation is show in panels C and D. The K562 cells were included as the positive control and the NK cells only as the negative control. Quantification from three experiments is shown in panels (E) and (F). *n* = 3 biological repeats. G) NK cells exhibited reduced lytic activity toward the uni OPCs, and this suppression was relieved partially by the treatment with a CD47 neutralizing antibody (*α*CD47). The luciferase reporter‐bearing WT or uni OPCs or the K562 cells that were included as a positive control were co‐cultured with NK cells for 48 h and the luciferase activity was measured. The uni OPCs exhibited much less lysis than K562 cells as revealed by the higher luciferase activity in the uni OPC than that of the K562 cells. The CD47 neutralization antibody increased NK‐mediated lysis of the uni OPCs as revealed by the reduced luciferase activity upon *α*CD47 treatment (uni OPC + *α*CD47). Error bars are SE of the mean. ns, not significant, *p < 0.05, **p < 0.01, and ***p < 0.001 by one‐way ANOVA followed by Tukey's multiple comparisons test for panels (E) and (F).

The immunogenicity of the universal OPCs was assessed both in vitro and in vivo. Because CD8^+^ cytotoxic T cells are the major effector cells of immune rejection, the effect of CD8^+^ cytotoxic T cells on universal OPCs was first assessed (**Figure** **6**A). The universal OPCs were co‐cultured with CD8^+^ T cells isolated from PBMC of multiple allogeneic donors. The WT OPCs and the allogeneic dendritic cells were included as the positive controls. The response of the T cells was evaluated by CellTrace T cell proliferation assay (Figure 6B; Figure S7A, Supporting Information). The universal OPCs evaded the response from allogenic CD8^+^ T cells. In contrast, both the WT OPCs and the allogenic dendritic cells stimulated CD8^+^ T cell proliferation and generated a reactive CD8^+^ T cell population (Figure 6B; Figure S7A, Supporting Information). Next, OPC‐specific reactive CD8^+^ T cells were isolated from co‐culture of the WT OPCs and T cells. These OPC‐specific T cells were used to treat the WT or universal OPCs to determine the cytotoxicity of the CD8^+^ T cells (**Figure** **7**A). The universal OPCs could evade the cytolytic activity from the reactive CD8^+^ cytotoxic T cells, whereas the WT OPCs were lysed by the T cells as revealed by dramatically reduced cell number of co‐cultured WT OPCs (Figure 6C; Figure S7B, Supporting Information) and substantially decreased luciferase activity in WT OPCs that carry a luciferase reporter, compared to that of the universal OPCs (Figure 6D; Figure S7C, Supporting Information). The immunogenicity of the universal OPCs against the CD4^+^ helper T cells was evaluated similarly by the CellTrace T cell proliferation assay. The universal OPCs or the WT OPCs were co‐cultured with CD4^+^ helper T cells derived from the PBMC of allogeneic donors. While the WT OPCs stimulated a proliferated CD4^+^ T cell population, the universal OPCs failed to do so (Figure 6E; Figure S7D, Supporting Information).

Next, we determined the effect of NK cells on universal OPCs. A previous study showed that CD47 could partially suppress NK cell activity.^[^ ^10b^ ^]^ Of interest to us, we found that OPCs naturally expressed high level of CD47, while iPSCs did not exhibit detectable CD47 expression (Figure 7A,B). We first determined the effect of NK cells on universal OPCs by the degranulation assay. The expression of CD107a, a marker of NK cell activation and cytotoxic degranulation, was assessed after incubating NK cells with the WT OPC or the universal OPCs. K562 cells, a known NK target, were included as a positive control. NK cells incubated with both the WT OPCs and the universal OPCs exhibited substantially lower expression of CD107a than NK cells incubated with K562 (Figure 7D,F; Figure S8C,D, Supporting Information). Although NK cells incubated with the universal OPCs exhibited slightly higher expression of CD107a compared to NK cells incubated with the WT OPCs, the difference did not reach statistical significance (Figure 7D,F; Figure S8C,D, Supporting Information). In contrast, the universal iPSCs exhibited higher level of CD107a compared to the WT iPSCs when incubated with NK cells (Figure 7C,E; Figure S8A,B, Supporting Information). Accordingly, NK cells exhibited reduced cell lytic activity toward the universal OPCs compared to the lytic activity toward K562 in a luciferase reporter assay (Figure 7G). This suppression of NK lytic activity could be relieved partially by treating the universal OPCs with a CD47‐neutralizing antibody (Figure 7G). These results together indicate that CD47 expressed on OPCs helps to suppress NK cell activity on universal OPCs.

### 2.8 Universal OPCs Exhibit Low Immunogenicity In Vivo

**Figure 8:**
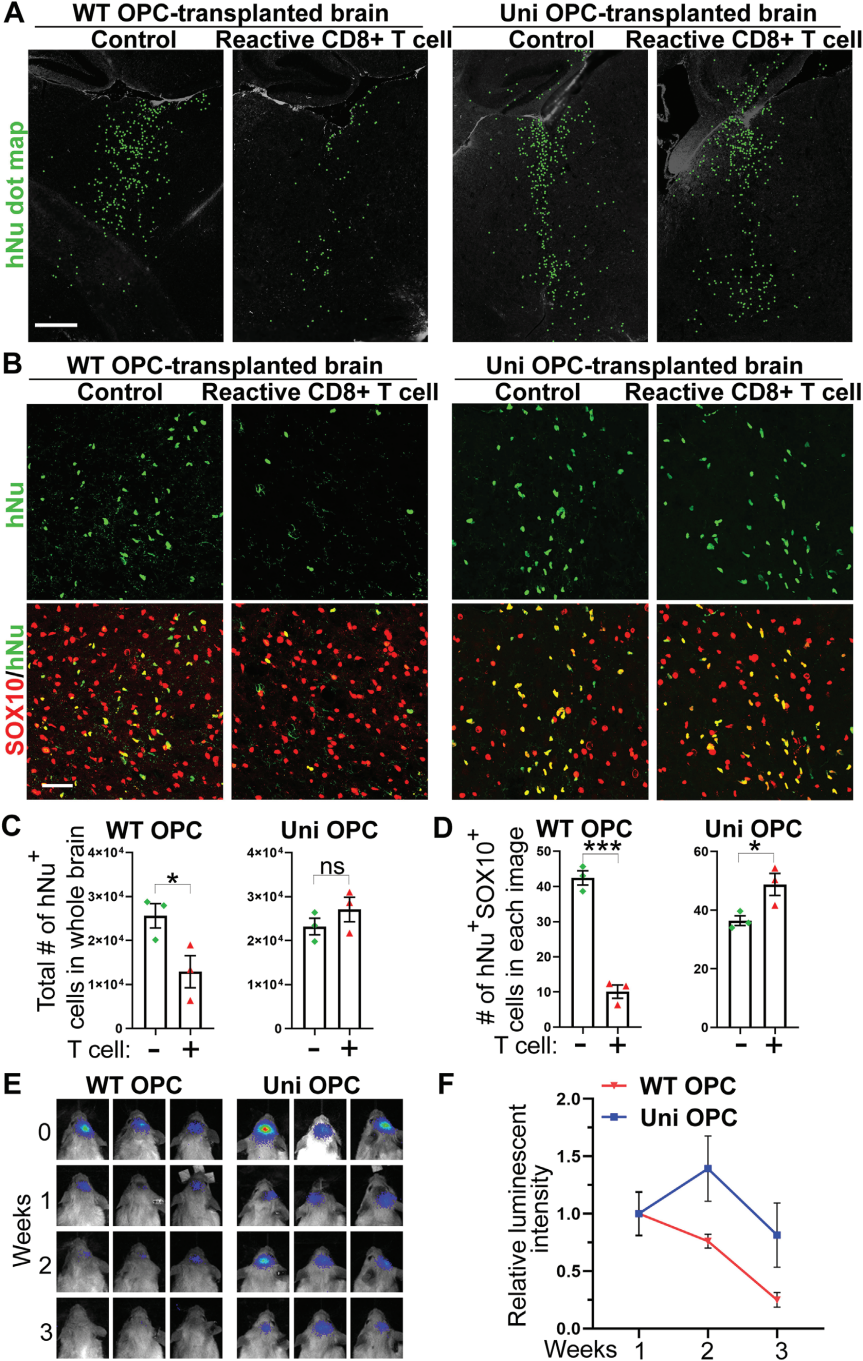
The universal (uni) OPCs exhibit low immunogenicity in in vivo. A,C) The uni OPCs escaped immune response from specific CD8^+^ reactive T cell in CD mice. The WT‐OPC‐specific CD8^+^ reactive T cells were mixed with WT OPCs or uni OPCs and transplanted into the brains of CD pups. WT and uni OPCs without mixing with the T cells were injected in parallel as the controls. The dot map of hNu staining is shown in panel (A). The quantification of hNu^+^ cells in the whole brain was shown in panel (C). *n* = 3 mice for each group. Scale bar: 500 µm. B,D) The uni OPCs survived better than WT OPCs in brains of CD mice transplanted with reactive CD8^+^ T cells. Co‐staining of CD mouse brains transplanted with WT or uni OPCs plus CD8^+^ T cells for hNu and SOX10. Scale bar: 50 µm. The quantification of hNu^+^SOX10^+^ cells in different groups is shown in panel (D). Three images were taken from each brain and hNu^+^SOX10^+^ cells in each image were counted. The average of three images from one brain was shown as one dot. *N* = 3 mice for each group. E,F) The uni OPCs escaped immune response from human PBMC in a humanized mouse model reconstituted with human PMBC. The bioluminescence images of NSG mice transplanted with WT or uni OPCs followed by allogenic PBMC injection are shown in panel (E), and the quantification of the relative bioluminescence intensity is shown in panel (F). Error bars are SE of the mean for panels (B) and (D). ns, not significant, *p < 0.05, ***p < 0.001 by Student's *t*‐test (two tailed).

To assess the immunogenicity of the universal OPCs in vivo, human CD8^+^ reactive T cell‐engrafted humanized CD mice were used. For this purpose, the WT or universal OPCs were transplanted into the brains of Aspa^(Nur7/Nur7)/^Rag2^−/−^ CD mice along with the OPC‐reactive CD8^+^ T cells (the humanized CD mice). OPC transplantation into the Aspa^(Nur7/Nur7)^/Rag2^−/−^ immunodeficient CD mice in the absence of the human CD8^+^ reactive T cells were included as a control. The survival of the transplanted WT or universal OPCs in the humanized CD mice (with human CD8^+^ reactive T cells) and the immunodeficient CD mice (without human CD8^+^ T cells) was compared after 14 days of transplantation by immunostaining for hNu or hNu and SOX10. The hNu^+^ cells or hNu^+^SOX10^+^ human OPCs were reduced substantially in the WT OPC‐transplanted humanized CD mice with human CD8^+^ reactive T cell engraftment, compared to that in the WT OPC‐transplanted immunodeficient CD mice without human T cell engraftment (**Figure** **8**A–D). In contrast, there was no significant decrease in the number of hNu^+^ cells or hNu^+^SOX10^+^ human OPCs in universal OPC‐transplanted CD mice engrafted with or without the human CD8^+^ reactive T cells (Figure 8A–D). The observation that universal OPCs could survive in the humanized CD mice (with human reactive T cells) to an extent similar to that in the immunodeficient CD mice (Figure 8A–D) indicates that the universal OPCs could evade T cell rejection.

To further monitor the effect of human immune cells on universal OPCs, we engrafted human PBMC through tail vein injection into NSG mice that allow better survival of human immune cells than Rag2^−/−^ mice.^[^ ^25^ ^]^ The WT or universal OPCs carrying a luciferase reporter gene were transplanted into the brains of humanized NSG mice that were generated by infusing human PBMC through tail vein injection. The survival of the transplanted OPCs was monitored by luminescence imaging. Mice transplanted with the WT OPCs exhibited dramatically reduced luminescent signal along a 3‐week time course (Figure 8E,F), indicating that the WT OPCs were rejected with time. Of note, mice transplanted with the universal OPCs exhibited higher luminescence intensity than mice transplanted with the WT OPCs along the time course (Figure 8E,F). These results indicate that the universal OPCs exhibit reduced immunogenicity compared to the WT OPCs and can escape immune surveillance in vivo. These results, together with the results from the human CD8^+^ reactive T cell‐engrafted humanized CD mice (Figure 8A–D), indicate that the universal OPCs can survive well in the humanized mouse models engrafted with human immune cells, unlike the WT OPCs that survived poorly in the humanized mouse models. Therefore, the universal OPCs could provide a cell source of hypoimmunogenic nature for allogeneic cell therapy development for myelin disorders.

## 3 Discussion

CD is a severe, progressive leukodystrophy that has no cure. In this study, we developed “off‐the‐shelf” hypoimmunogenic OPCs from healthy donor‐derived iPSCs carrying endogenous WT *ASPA* gene as promising therapeutic candidates for CD. To generate an “off‐the‐shelf” cell product that can evade the immune surveillance, we first disrupted HLA‐I and HLA‐II expression in iPSCs and differentiated the engineered iPSCs into hypoimmunogenic OPCs. These hypoimmunogenic OPCs exhibited reduced immunogenicity both in vitro and in vivo. WT OPCs were differentiated from healthy donor‐derived iPSCs that were not genetically engineered and included as a control. The hypoimmunogenic OPCs were transplanted into the brains of CD (nur7) mice. The transplanted OPCs could survive well and migrate widespread into the whole brains. Moreover, the transplanted hypoimmunogenic OPCs could mature into oligodendrocytes and express endogenous *ASPA* mRNA and protein that is enzymatically active. The reconstituted ASPA enzyme was able to improve the disease symptoms dramatically, as revealed by decreased NAA levels, substantially reduced spongy degeneration and rescued myelination in the transplanted brains, and restored motor functions in the transplanted mice. The hypoimmunogenic OPCs were able to rescue the CD phenotypes in a manner that is comparable to the WT OPCs.

“Off‐the‐shelf” allogenic universal donor cells are an attractive cell source because a single master cell bank can be generated and used for multiple patients and a series of related diseases. In this study, we created hypoimmunogenic universal donor OPCs by impairing HLA‐I and HLA‐II expression in iPSCs derived from a healthy donor. The hypoimmunogenic OPCs exhibited effective escape from the immune response elicited by allogenic T cells in vitro and in vivo, consistent with the observations from previous studies.^[^ ^9^, ^10^, ^11^ ^]^ Moreover, we found that the highly specialized OPCs could suppress NK activation partially in the absence of HLA‐I protection. CD47 has been reported to be effective at inhibiting NK cell‐mediated cytotoxicity against universal PSC‐derived grafts.^[^ ^10b^ ^]^ Our OPCs naturally express CD47, which may contribute to the suppressive effect on NK attack. OPCs may also express other immune suppressors. Multiple reports have shown that overexpression of HLA‐E, HLA‐G or knock out of activating ligands of NK cells suppressed cytotoxicity from different NK population.^[^ ^10^, ^11^, ^13^, ^26^ ^]^ In the future, these hypoimmunogenic iPSCs can be further modified by including more immune suppressors to better resist NK cell‐mediated attack.

Studies have been designed to treat CD by using in vivo gene therapy or *ex vivo* gene‐cell combined therapy.^[^ ^1^, ^8^, ^15^ ^]^ The unique advantage of this study is to reconstitute endogenous WT *ASPA* expression in transplanted oligodendrocytes. Previous studies have used generic or cell type‐specific exogenous (non‐ASPA) promoter to drive *ASPA* expression. However, studies have shown that lower level of NAA could cause neurological deficits including abnormal neuronal recruitment and startle deficits.^[^ ^3^, ^27^ ^]^ Therefore, maintaining NAA at the physiological level is critical for proper brain functions. Expression of the *ASPA* gene under the endogenous *ASPA* promoter in oligodendrocytes will ensure that the expression level of the ASPA enzyme under control.

In the early stage of gene therapy for CD, AAV2‐ASPA with neuronal tropism was used to treat CD patients in a clinical trial.^[^ ^17^, ^28^ ^]^ However, the therapeutic effects were limited.^[^ ^17b^ ^]^ Because ASPA is mainly expressed in oligodendrocytes in the human brain, the ASPA transgene was delivered using a new generation AAV that was developed with tropism for oligodendrocytes,^[^ ^17c,d^ ^]^ or the *ASPA* transgene was placed under the control of an oligodendrocyte‐specific promoter, such as the MBP or the MAG promoter, to drive ASPA expression in oligodendrocytes.^[^ ^1b^ ^]^ The vector AAV/Olig001 with oligodendrocyte tropism exhibited high transduction efficiency in oligodendrocytes. However, a certain population of neurons were still infected, at an efficiency that was even higher than that in oligodendrocytes in the cerebellum.^[^ ^17c,d^ ^]^ For oligodendrocyte‐specific promoter, the human MAG promoter and the mouse MBP promoter have exhibited specificity for oligodendrocytes in mouse brains but with leaky expression in neurons and astrocytes.^[^ ^3^, ^29^ ^]^ In this study, only oligodendrocytes derived from the engrafted OPCs expressed ASPA. Therefore, our therapeutic strategy allows the control of ASPA expression in the right cell type (oligodendroglial cells) at the physiological level (driven by the endogenous promoter).

The clinical trial using AAV2‐ASPA reduced elevated NAA level in CD patient brains but had limited therapeutic outcomes,^[^ ^17b^ ^]^ suggesting that reducing NAA level alone in CD patient brains may not be sufficient to repair damaged myelin structures. Besides providing a source for the ASPA enzyme, the transplanted OPCs can be matured into oligodendrocytes to supply myelin for remyelination. Moreover, it has been shown that high level of NAA fails to destroy myelin from oligodendrocytes expressing a functional ASPA enzyme,^[^ ^3^, ^30^ ^]^ therefore the engrafted OPC‐derived oligodendrocytes that express a functional ASPA enzyme can better resist the toxicity from any residual or fluctuated elevated NAA level.

## 4 Conclusion

Demyelinating diseases of the central white matter are among the most prevalent and disabling neurological disorders.^[^ ^31^ ^]^ Human iPSC‐derived OPCs can give rise to oligodendrocytes for the restoration of myelin in demyelinated brains. OPCs can also be used as an efficient delivery vector for endogenous or exogenous medicine to treat brain diseases, such as metabolic disorders in the brain. For example, OPCs derived from iPSCs of a healthy donor can be used to provide the ASPA enzyme for CD or the galactosylceramidase enzyme for Krabbe disease. The “off‐the‐shelf” hypoimmunogenic OPCs can be used as universal donor cells to treat multiple patients of various demyelinating diseases, including Alzheimer's disease and multiple sclerosis. In summary, this study provides a promising therapeutic candidate for CD and many other demyelinating diseases.

## 5 Experimental Section

### Immunodeficient CD (Nur7) Mice and NSG Mice

All animal housing condition and surgical procedures were approved by and conducted according to the Institutional Animal Care and Use Committee of City of Hope (IACUC#03038). Immunodeficient CD (Nur7) mice had been characterized and used in the previous study.^[^ ^8^ ^]^ Briefly, *ASPA^nur7/+^* (*ASPA^nur7^*/J, 008607) and *Rag2* ^−/‐^ mice (B6(Cg)‐*Rag2^tm1.1Cgn^*/J, 008449) were purchased from the Jackson Laboratory and crossed to generate double knockout mice. The *ASPA^nur7/nur7^*/*Rag2^−/‐^* mice were called CD (Nur7) mice for transplantation and negative control. The *ASPA^+/+^*/*Rag2^−/‐^* mice and *ASPA^nur7/+^*/*Rag2^−/‐^* mice were respectively called WT and Het mice as positive control. NSG mice (NOD.*Cg‐Prkdc^scid^Il2rg^tm1Wjl^*/SzJ 008607) were purchased from the Jackson Laboratory to create PBMC‐humanized mouse model.

### Generation of the WT and Hypoimmunogenic iPSCs

Human healthy donor iPSCs were generated from I90 fibroblasts (IMR90, female, Coriell). The resultant I90 iPSCs were termed WT iPSCs because they carry the WT *ASPA* gene. These cells were characterized in the previous study.^[^ ^15e^ ^]^ CRISPR‐Cas9 editing was used to generate hypoimmunogenic iPSCs from the WT iPSCs. The editing was performed using an RNP (ribonucleoprotein) complex consisted of the sgRNA targeting the *B2M* gene, 5′‐ CGT GAG TAA ACC TGA ATC TT‐3′, the sgRNA targeting the *CIITA* gene, 5′‐GAT ATT GGC ATA AGC CTC CC‐3′, and the recombinant Cas9 protein. The RNA complex was transfected into WT iPSCs using the 4D‐Nucleofector (Lonza).^[^ ^10b^ ^]^ After electroporation, single‐cell colonies were developed. The iPSC colonies were screened by PCR and Sanger sequencing. The B2M primers are forward: 5′‐TGG GGC CAA ATC ATG TAG ACT C‐3′ and reverse: 5′‐TCA GTG GGG GTG AAT TCA GTG T‐3′. CIITA primers are forward: 5′‐CTT AAC AGC GAT GCT GAC CCC‐3′ and reverse: 5′‐TGG CCT CCA TCT CCC CTC TCT T‐3′. Four clones with frameshift for both the *B2M* and the *CIITA* genes were selected for further characterization. One of the clones was selected randomly for OPC differentiation and further experiments.

### Differentiation of hiPSCs into OPCs

The WT and hypoimmunogenic iPSCs were used to generate OPCs by following a previously published protocol.^[^ ^8^, ^15^ ^]^ Generally, iPSCs that were dissociated into single‐cell suspensions were seed on Matrigel‐coated plates one day before induction. Then cells were switched to OPC‐I medium containing DMEM/F12 (Thermo Fisher, 11330032), 1x N2 (Thermo Fisher, 17502048), 1x NEAA (Gibico, 11140076), 2 mm GlutaMAX (Thermo Fisher, 35050061), 0.1 µm Retinoic acid (RA, Sigma, R2625), 10 µm SB431542 (Peprocell, 04‐0010‐10), and 250 nm LDN‐193189 (Peprocell, 04‐0074‐10) for 8 days to induce neural progenitor cells. From day 8 to day 11, attached cells were switched to OPC‐II Medium containing DMEM/F12, 1x N2, 1x NEAA, 2 mm GlutaMAX, 0.1 µm RA and 1 µm SAG (Sigma, ML1314) to induce OLIG2‐positive pre‐OPCs. On day 12, attached pre‐OPCs were dissociated and switched to OPC‐III Medium containing DMEM/F12, 1x N2, 1x B27 minus vitamin A (Thermo Fisher, 12587010), 1x NEAA, 2 mM GlutaMAX, 0.1 µM RA and 1 µM SAG to form spheres in suspension culture in flasks from day 12 to 19, and then switched to PDGF medium containing DMEM/F12, 1x N2, 1x B27 minus vitamin A, 1x NEAA, 2 mm GlutaMAX, 10 ng mL^−1^ PDGF‐AA (R&D, 221‐AA‐050), 10 ng mL^−1^ IGF‐1 (R&D, 291‐GG‐01M), 5 ng mL^−1^ HGF (R&D, 294‐HG‐250), 10 ng mL^−1^ NT3 (EMD Millipore, GF031; and PeproTech, AF‐450‐03), 60 ng mL^−1^ T3 (Sigma, T2877), 100 ng mL^−1^ Biotin (Sigma, 4639), 1 µm cAMP (Sigma, D0627), and 25 µg mL^−1^ Insulin (Sigma, I9278) from day 20 to 29. From day 30, spheres were attached on Matrigel‐coated plates in PDGF medium for OPC generation. Live staining of O4 was used to monitor differentiation. O4‐positive OPCs were first found at day 50–60. After 70 days of differentiation, extensive O4‐positive OPCs were generated, and the differentiated cells were ready for transplantation and analysis.

### O4 Live Staining

Live staining of O4 was used to monitor the OPC differentiation process. The primary O4 antibody was added into the culturing medium and incubated in the tissue culture incubator at 37 °C, 5% CO2 for 30 min. After removing the medium and washing with 1 mL of DMEM/F12, new culture medium containing the anti‐mouse IgM secondary antibody was added and incubated for 30 min in the tissue culture incubator. Then medium was removed and replaced with new culture medium. The O4 signal was monitored under the microscope.

### Flow Cytometry Analysis

iPSCs and OPCs were dissociated with accutase to obtain single‐cell suspension for staining. Immune cells isolated from PBMCs were directly used for staining. DPBS containing 2% FBS was used as the washing and staining buffer. For staining, cells were suspended and incubated with 50 µL of diluted antibodies for 15–20 min on ice. For O4 staining, cells were washed after primary antibody incubation and stained by secondary antibody for 15–20 min on ice. Samples were washed, resuspended in buffer with DAPI and analyze on Attune NxT Flow Cytometer (ThermoFisher Scientific). No DAPI was added if cells were labeled by CellTrace‐violet. The data were analyzed by FlowJo v10. The detailed information of all the primary antibodies and isotype controls used were listed in Table S1 (Supporting Information).

### Immunocytochemistry

Differentiated OPCs were dissociated with accutase and seed on Matrigel‐coated chamber slides (Ibidi, 80826) for immunostaining. After 2–7 days of culturing, cells were first stained with the O4 primary antibody following the O4 live staining protocol described above if need. Then cells were taken out and fixed with 4% paraformaldehyde (PFA) for 10 min, permeabilized and blocked with 5% donkey serum diluted in PBS with 0.1% triton (PBST) for 1 hour at RT. The fixed cells were then incubated with primary antibodies at 4 °C for overnight. On the following day, cells were washed and incubated with secondary antibodies at RT for 1 hour. Cells were counterstained with DAPI before mounting for imaging. Images were taken using Nikon Ti‐2. For quantification, the well was divided into four equally parts and one image were taken for each part. Cells were counted manually, and the graphs were created by GraphPad Prism. The information of the primary antibodies and secondary antibodies was listed in Table S1 (Supporting Information).

### Stereotaxic Transplantation

Stereotaxic transplantation was performed as described previously.^[^ ^8^, ^32^ ^]^ Briefly, OPCs were dissociated into single cells with accutase and resuspend at 100 000 cells per µL in PDGF medium. PDGF medium only was injected as vehicle control. Postnatal day 1 to 4 (PND 1–4) mice were anesthetized on ice and cells were transplanted into six sites, 1.5 µL per site, in the brain by using two group of coordinates. For pups that weighed less than 2 g: the corpus callosum (+3.0, ±1.6, −1.3), the subcortical (0.5, ±1.0, −2.5), and the brain stem (−1.6, ±0.8, −3.0); for pups that weighed over 2 g: the corpus callosum (+3.5, ±1.7, −1.4), the subcortical (0.5, ±1.0, −2.5), and the brain stem (−1.6, ±1.0, −3.1). All the coordinates are (A, L, V) with reference to Lambda. “A” stands for antero‐posterior from Lambda, “L” stands for lateral from midline, and “V” stands for ventral from the surface of brain in Lambda, respectively.

### Serial Cryosectioning and Immunohistochemistry

Immunohistochemistry was performed as described previously.^[^ ^8^ ^]^ Briefly, mice were perfused and extracted brains were processed and embedded in OCT. Then the brains were serially cryosectioned at sagittal planes. Specifically, slides were first labeled. Serial sections were collected onto labeled slides with one section per slide, until all slides were used for collection. Repeat the procedure until all sections from a brain were collected. For immunohistochemistry analysis, brain sections were permeabilized and blocked with 5% donkey serum in PBST at RT for 1 h. Sections were then incubated with primary antibodies at 4 °C for overnight. Then sections were washed and incubated with secondary antibodies at RT for 1 h. Finally, slides were counterstained with Dapi, mounted with the mounting medium. The information of the primary antibodies and secondary antibodies was listed in Table S1 (Supporting Information). Confocal microscopy was performed on a Zeiss LSM 700 microscope (Zeiss) for imaging cell marker staining.

For cell fate quantification, images of transplanted cells in the white matter track including the corpus callosum, the subcortical and the brain stem regions, and the gray matter cortex were taken by Confocal microscopy. Total human cells and double positive cells in each image were counted for each brain. Three brains were analyzed in each group.

For dot map, cell counting in the whole brain and MBP quantification, sections were scanned by Nikon Ti‐2 or Zeiss Axioscan 7. Dot maps were made using Photoshop based on the hNu signal and the ASPA signal in the cell body. A serial sagittal dot maps were created. Each series was begun lateral to the midline and continued at 900 µm intervals. Qupath was used to count the hNu^+^ cells and the ASPA^+^ cells, and all sections from one representative slide were counted. The total number of the hNu^+^ cells and the ASPA^+^ cells was calculated. For MBP labeling quantification, three regions including the subcortical, the brainstem, and the white matter of the cerebellum, regions that have shown severe vacuolation were analyzed. The mean intensity and the percentage of the MBP^+^ area was measured using Image J. All sections from one representative slide of each brain were analyzed and at least three brains were analyzed for each mouse group.

### Measuring NAA Level and ASPA Activity in Brain Tissues

Fresh brains were directly extracted from mice, chopped, mixed well, aliquoted, and stored in −80 °C freezer. Two aliquots were taken and placed into two separate tubes. One tube was directly subjected to extraction to measure NAA level in the brain, while another tube was incubated at 37 °C followed by extraction to calculate ASPA activity using the difference of NAA level before and after 37 °C incubation. Perchloric acid (PCA, Sigma, 244252) method were used to extract aqueous metabolites from mouse brains as described.^[^ ^8^, ^15^, ^33^ ^]^ Samples were then subjected to NMR analysis at the NMR Core Facility of City of Hope. The NAA level was normalized with tissue weight and expressed as µmol per gram. The ASPA activity was calculated as the decreased NAA level in 1 gram of tissue within 1 h of incubation and expressed as µmol per gram of brain tissue per hour.

### Measuring NAA Level in the CSF

The CSF was collected as previously described with modifications.^[^ ^34^ ^]^ Briefly, mice were anesthetized by the Ketamine/xylazine cocktail. An incision was made above the neck and muscle and the atlanto‐occipital membrane was dissected to expose the cisterna magna. CSF was pulled out from the cisterna magna with home‐made pipette (Drummond Scientific, Broomall, PA, Cat. # 2‐000‐050, Green). The collected CSF samples were subjected to NMR analysis for NAA level at the NMR Core Facility of City of Hope.

### H&E Staining and Vacuolation Analysis

Hematoxylin and eosin (H&E) staining was performed at the Pathology Core of City of Hope. A one‐in‐eight series of whole brain slides from serial cryosection were stained and scanned under Nanozoomer HT (Hamamatsu Photonics, Japan) at the Light Microscopy Core of City of Hope. Three regions including the subcortical, the brainstem and the white matter of cerebellum, regions that have shown severe vacuolation were analyzed. The surface area of the vacuolated brain regions and the intact brain regions was measured using Image‐Pro Premier 9.2 for all sections. The percentage of vacuolation = [the area of vacuolated brain region / (the area of vacuolated brain region + the area of intact brain region)] × 100. All sections from one representative slide of each brain were analyzed and at least three brains were analyzed for each mouse group.

### Electron Microscopy (EM) and G‐Ratio Analysis of Myelin Sheaths

Mice were perfused by 0.1 m Millonig's buffer containing 4% paraformaldehyde (PFA) and 2.5% glutaraldehyde. Then brain tissues were extracted and processed at the Electron Microscopy and Atomic Force Microscopy Core of City of Hope. Brains were cut into ≈150 µm vibratome sections using a Leica VT 1000S vibratome. The heavy metal osmium tetroxide (OsO4) staining protocol developed by Dr. Mark Ellisman's group was followed for staining.^[^ ^35^ ^]^ Tiled vibratome brain section images were taken using Nikon Ti‐2. Then tissues from three regions of the brains including the subcortical, the brainstem and the white matter of the cerebellum were micro‐dissected and ultra‐thin sections were cut using a Leica Ultracut UCT ultramicrotome. Transmission electron microscopy was performed on an FEI Tecnai 12 transmission electron microscope equipped with a Gatan Ultrascan 2K CCD. More than three images were randomly taken for each sample in each region. The inner axonal diameter and the total outer diameter of more than 50 myelin sheathes in each region of the CD (Nur7) mice were measured using Image‐Pro Premier 9.2. Briefly, the outer edge of the inner axons (or inner edge of myelin sheaths) and the outer edge of the myelin sheaths were traced using the selection tool. The diameter of the axon and the outer diameter of the myelin sheaths were calculated by Image‐Pro. The g‐ratio is the ratio of the inner axonal diameter to the total outer diameter. The abnormal myelin sheaths were further identified based on the layer structure of the myelin sheaths which exhibited substantial difference between the Het and the CD (Nur7) mice.

### Rotarod Test and Grip Strength Test

The motor performance of mice was evaluated using a rotarod treadmill (Rotamex, Columbus Instruments) as described.^[^ ^8^, ^15^, ^19^ ^]^ Briefly, mice were tested for the latency on the rod when the rod was rotating at the accelerating speed (2–65 rpm) in a 2‐min trial session. Each mouse was monitored for the latency 4 times per test. The average time was calculated and showed as one dot in graph. The forelimb strength of mice was measured using a grip strength meter (BIO‐GS3, Bioseb) to detect motor coordination and motor function as described.^[^ ^8^, ^15^ ^]^ The grip strength of the mouse was recorded by gently pulling the tail of the mouse backward until release. Four sequential measurements were performed, and the average strength was calculated and showed as one dot in graph. At least 6 mice for each group were tested.

### qRT‐PCR

Mouse brain samples were aliquoted from chopped and mixed brains. Human cortex brain tissues were obtained from Banner Sun Health Research Institute. The evaluation from our Institutional Review Board determined that research with these coded tissues without identifiers from deceased subjects do not meet the definition of human subject research. Total RNAs were extracted using TRIazol (Invitrogen, 15596018). 1 µg of RNA was reverse transcribed into cDNA using the Tetro cDNA synthesis kit (Bioline, BIO‐65043). Real‐time PCR was performed using DyNAmo Flash SYBR Green qPCR mix on a StepOnePlus system (Applied Biosciences). Human‐specific *ASPA* primers (forward: 5′‐CAC TAC CCT GCT ACG TTT ATC TG‐3′ and reverse: 5′‐GGA TAC TTG GCT ATG GAA CGA G‐3') were designed and evaluated in mouse and human brains. The *β*‐actin primers (forward: 5′‐GGC TTC GCG GGC GAC GAT GC‐3′ and reverse: 5′‐CTC TCT TGC TCT GGG CCT CGT C‐3') that are good for both mouse and human were designed, and the PCR product of *β*‐actin was used as the loading control.

### CellTrace Proliferation Assay for CD8^+^ T Cells

Peripheral blood samples from de‐identified healthy donors were obtained from the Michael Amini Transfusion Medicine Center of City of Hope under institutional review board‐approved protocols. PBMCs were isolated with Ficoll and stored in liquid nitrogen. After thawing, PBMCs were cultured in RPMI‐1640 containing GlutaMAX and 10% heat‐inactivated human serum (Valley Biomedical, HP1022HI) for overnight. On the following day, CD8^+^ T cells were isolated from PBMC by using CD8^+^ T cell isolation kit (StemCell, Cat. 17953) and labeled with CellTrace‐Violet (Invitrogen, C34557) or CellTrace‐FarRed (Invitrogen, C34572). At the same time, OPCs pretreated with 50 ng mL^−1^ IFN‐*γ* for 48 h, were dissociated with accutase and irradiated with 35 Gy to stop proliferation. The labeled CD8^+^ T cell and irradiated OPCs were co‐cultured at a 1:1 ratio in a 96‐well U‐bottom plate for 7 days in RPMI‐1640 containing GlutaMAX, 10% heat‐inactivated human serum and 25 U mL^−1^ IL‐2. Monocytes were enriched from allogenic PBMCs (StemCell, Cat. 19059) and differentiated into Dendritic cells as positive targeting cells. The CD8^+^ T cell became Violet or FarRed negative after proliferation and the percentage was measured by flow cytometry. To have enough reactive CD8^+^ T cell for cytotoxicity assay CD8^+^ T cells isolated from PBMCs were co‐cultured with the WT OPCs in a 24‐well plate for 7–14 days. The CD8^+^ T cells were isolated from the co‐cultures by using the CD8^+^ T cell isolation kit for cytotoxicity assay.

### Luciferase Assay for Evaluating the Cytotoxicity of Reactive CD8^+^ T Cells

For luciferase assay, OPCs that expressed a luciferase reporter and were pretreated with 50 ng mL^−1^ IFN‐*γ* for 48 h were dissociated with accutase, irradiated with 35 Gy and seeded on a 96‐well micro plate with flat‐bottom (Greiner Bio‐One, 655090) one day before co‐culture. On the following day, the isolated T cells with reactive CD8^+^ T cells were labeled with CellTrace‐FarRed and added into the wells in a 96‐well plate that were pre‐seeded with OPCs one day before with different ratio. After 2 days of culturing, images were taken by Nikon Ti‐2. Then luciferase activity in different wells were analyzed using the ONE‐Glo Luciferase Assay System (Promega, E6120).

### CellTrace Proliferation Assay for CD4 T Cells

Isolated PBMCs were thawed in RPMI‐1640 containing GlutaMAX, 10% heat‐inactivated human serum and 100 U mL^−1^ IL‐2 for overnight. On the following day, the CD4^+^ T cells were isolated from PBMC by using the CD4^+^ T cell isolation kit (StemCell, Cat. 17952) and labeled with CellTrace‐FarRed. At the same time, OPCs, pretreated with 50 ng mL^−1^ IFN‐*γ* for 48 h, were dissociated and irradiated with 35 Gy to stop proliferation. The labeled CD4^+^ T cell and irradiated OPCs were cocultured at a 1:1 ratio in a 96‐well U‐bottom plate for 7 days in RPMI‐1640 containing GlutaMAX, 10% heat‐inactivated human serum and 25 U mL^−1^ IL‐2. The CD4^+^ T cells became FarRed‐negative after proliferation and the percentage was measured by flow cytometry.

### CD107a Degranulation Assay for NK Cells

OPCs, pretreated with 50 ng mL^−1^ IFN‐*γ* for 48 h, were dissociated, irradiated with 35 Gy and seeded onto a 96‐well U‐bottom plate one day before co‐culture. K562 cells were irradiated and seeded as the positive control. Isolated PBMCs were thawed in RPMI‐1640 containing GlutaMAX, 10% heat‐inactivated human serum and 100 U mL^−1^ IL‐2 for overnight. Then NK cells were enriched with the NK cell enrichment kit (StemCell, Cat. 19055) and added into wells that were pre‐seeded with iPSCs, OPCs or K562 cells for 5 h at a 2.5:1 E:T ratio in RPMI‐1640 containing GlutaMAX, 10% heat‐inactivated human serum, 1:50 diluted CD107a antibody, 100 U mL^−1^ IL‐2 for OPCs and K562 co‐culturing and 500 U mL^−1^ IL‐2 for iPSCs co‐culturing. Finally, the percentage of CD107a‐positive NK cells was measured by flow cytometry analysis.

### Luciferase Assay for Cytotoxicity of NK Cells

OPCs expressing luciferase, pretreated with 50 ng/mL IFN‐*γ* for 48 hours, were dissociated, irradiated with 35 Gy and seed onto a 96‐well plate with flat bottom one day before coculturing. K562 cells transduced with luciferase virus were irradiated and seed as positive control. Isolated PBMCs were thawed in RPMI‐1640 containing GlutaMAX, 10% heat‐inactivated human serum and 50 U/mL IL‐2 for overnight. Then NK cells were enriched with NK cell enrichment kit and added into wells with OPCs or K562 cells with series ratio for 48 hours in RPMI‐1640 containing GlutaMAX, 10% heat‐inactivated human serum and 50 U/mL IL‐2. Finally, luciferase activity in different wells were analyzed with ONE‐Glo™ Luciferase Assay System.

### In Vivo Immunogenicity in Human PBMC‐Grafted NSG Mice

OPCs expressing a luciferase reporter were dissociated into single cells and transplanted into the frontal lobe of NSG mouse brains by stereotaxic intracranial injection as previously described.^[^ ^36^ ^]^ Isolated PBMCs were thawed in RPMI‐1640 containing GlutaMAX, 10% heat‐inactivated human serum for overnight. PBMCs were injected into OPCs‐transplanted NSG mice through tail vein injection on the second day. The survival of OPCs was monitored by bioluminescence imaging every week. The bioluminescence intensity was quantified.

### In Vivo Immunogenicity in Reactive CD8 T Cell‐Grafted CD Mice

The WT‐OPC‐reactive CD8^+^ T cells were first expanded in the T cell expansion medium consisting of RPMI‐1640, GlutaMAX, 10% heat‐inactivated human serum, 50 U mL^−1^ IL‐2, 10 ng mL^−1^ IL‐7, and 10 ng mL^−1^ IL‐15. For transplantation, OPCs were dissociated into single cells and equally divided into two groups. In the control group, OPCs were directly resuspended at 25 000 cells per µL in the T cell expansion medium. In the T cell‐treated group, OPCs were mixed with reactive CD8^+^ cells at 1:1 ratio, 25 000 OPC cells and 25 000 T cells per µL in the T cell expansion medium. Then, cells from the two groups were transplanted in parallel into the brains of Postnatal day 1 to 4 (PND 1–4) CD mice by using the stereotaxic transplantation method as described above. The subcortical coordinates (0.5, ±1.0, −2.5) were used for transplantation. 14 days after transplantation, the brains were collected and serially sectioned. One slide of every 8 slides, with total 4 slides from a total of 32 slides, were stained by hNu antibody and the hNu^+^ cells were counted by Qupath. The total cell number was calculated and shown. For imaging and quantification of hNu^+^SOX10^+^ OPCs, the slides were stained by the hNu and SOX10 antibodies and imaged by Zeiss LSM 900 confocal microscope (Zeiss). Three images were taken for each mouse brain and the cell number was counted and shown.

### Statistical Analyses

Data are shown as means ± SE as specified in the figure legends and analyzed with GraphPad Prism 9 (San Diego, CA). The number of mice analyzed per treatment group is indicated as “n” in the corresponding figure legends. No exclusion criteria were applied. Animals were assigned randomly to treatment groups. The study was not blinded. Student's *t*‐test (two‐tailed), one‐way ANOVA followed by Dunnett's multiple comparisons test or Tukey's multiple comparisons test, and two‐way ANOVA followed by Benferroni's multiple comparisons test and by Šidák's multiple comparisons test were used for statistical analysis as reported in each figure legend. *p* < 0.05 was considered statistically significant. *p < 0.05, **p < 0.01, and ***p < 0.001.

## Conflict of Interest

The authors declare no conflict of interest.

## Author Contributions

L.F. and Y.S. designed the study. L.F. designed and performed majority of the experiments. J.C. prepared brain samples for NAA and ASPA activity measurement. P.Y. performed animal‐related experiments including surgery, behavior tests, tissue collection and sectioning. Q.L. and S.F. analyzed part of collected brains by immunostaining and quantification. G.S. did cell irradiation. W.L. prepared cDNA from mouse brains and performed qRT‐PCR analysis. Q.C. offered luciferase analysis system for in vitro and in vivo experiments. N.J., A.N.H.S., and G.S. performed part of the brain sectioning. Z.L. prepared cDNA from human cortex. W.H. measured concentration of NAA. L.F. and Y.S. prepared the manuscript with input from all other authors.

## Supporting information

Supporting Information

## Data Availability

The data that support the findings of this study are available from the corresponding author upon reasonable request.
