# Supplementary material for: Developing Hypoimmunogenic Human iPSC‐Derived Oligodendrocyte Progenitor Cells as an Off‐The‐Shelf Cell Therapy for Myelin Disorders
Source: Adv Sci (Weinh). 2023 Jun 4;10(23):2206910. doi: 10.1002/advs.202206910 (PMC10427412; doi:10.1002/advs.202206910)
Supplement: Supplementary file 1 — Supporting Information [file ADVS-10-2206910-s001.pdf]

ADVANCED SCIENCE
Open Access

Supporting Information

for *Adv. Sci.*, DOI 10.1002/advs.202206910

Developing Hypoimmunogenic Human iPSC-Derived Oligodendrocyte Progenitor Cells as an Off-The-Shelf Cell Therapy for Myelin Disorders

*Lizhao Feng, Jianfei Chao, Peng Ye, Qui Luong, Guoqiang Sun, Wei Liu, Qi Cui, Sergio Flores, Natasha Jackson, Afm Nazmul Hoque Shayento, Guihua Sun, Zhenqing Liu, Weidong Hu and Yanhong Shi**

Supporting Information

Developing off-the-shelf hypoimmunogenic human iPSC-derived oligodendrocyte progenitor cells as an allogeneic cell therapy for myelin disorders

*Lizhao Feng, Jianfei Chao, Peng Ye, Qui Luong, Guoqiang Sun , Wei Liu, Qi Cui, Sergio Flores, Natasha Jackson, Afm Nazmul Hoque Shayento, Guihua Sun, Zhenqing Liu, Weidong Hu, Yanhong Shi**

Supplementary Figures and Legends

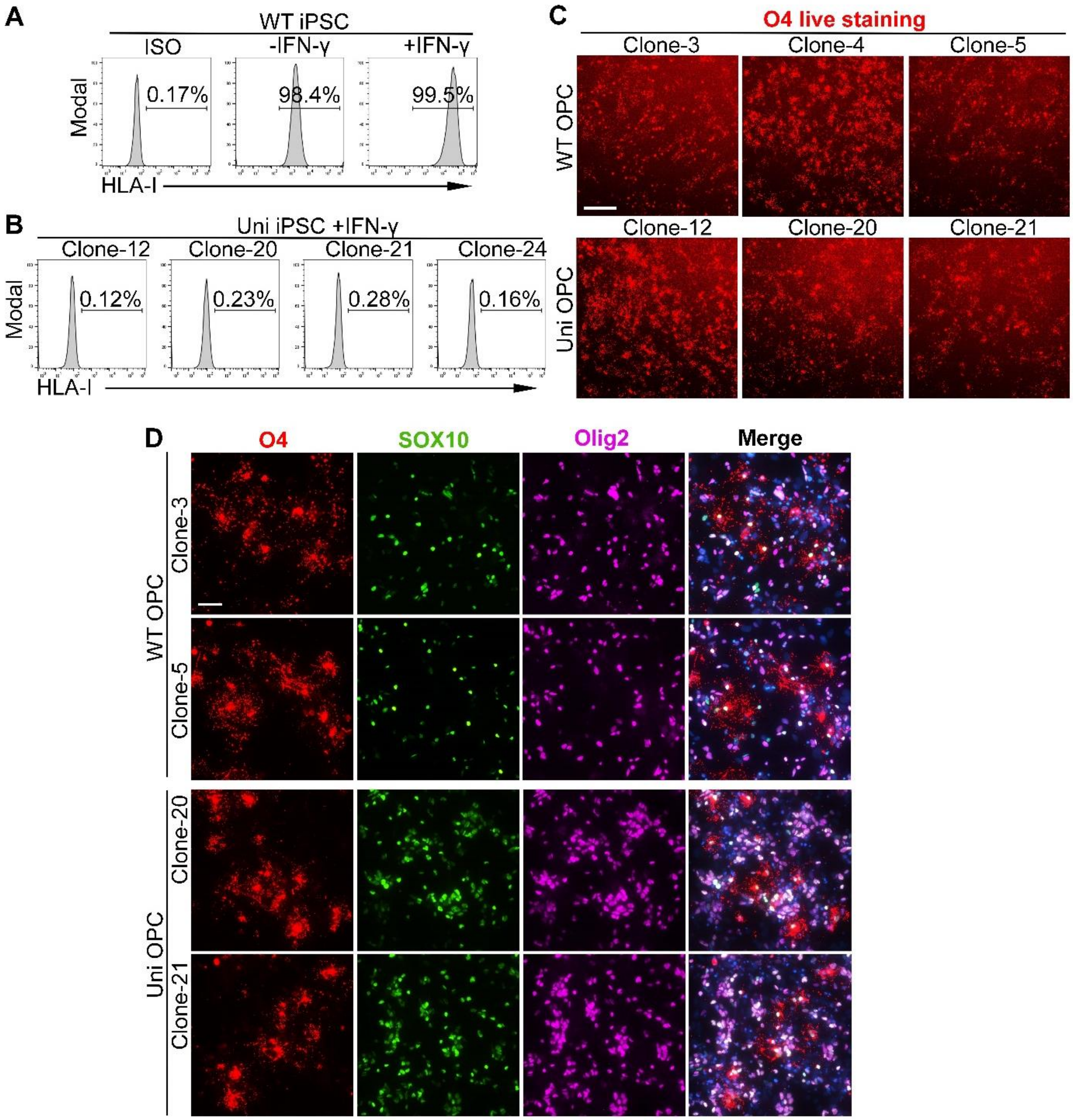

Figure S1. Characterization of the universal (uni) iPSCs and OPCs, related to Figure 1. (A) The WT iPSCs expressed HLA-I and the expression was enhanced after IFN-γ treatment. Flow cytometry analysis of the WT iPSCs with the HLA-I antibody. The isotype IgG was included as the negative control. (B) The uni iPSCs lost the expression of HLA-I even with IFN-γ treatment after knockout of the *B2M* gene. (C) Live staining of O4 after 70 days of differentiation for multiple clones from WT and uni OPCs. Scale bar: 100 µm. (D) Immunostaining of multiple clones from WT and uni OPCs for the oligodendroglial lineage markers O4, SOX10, and OLIG2. Scale bar: 50 µm.

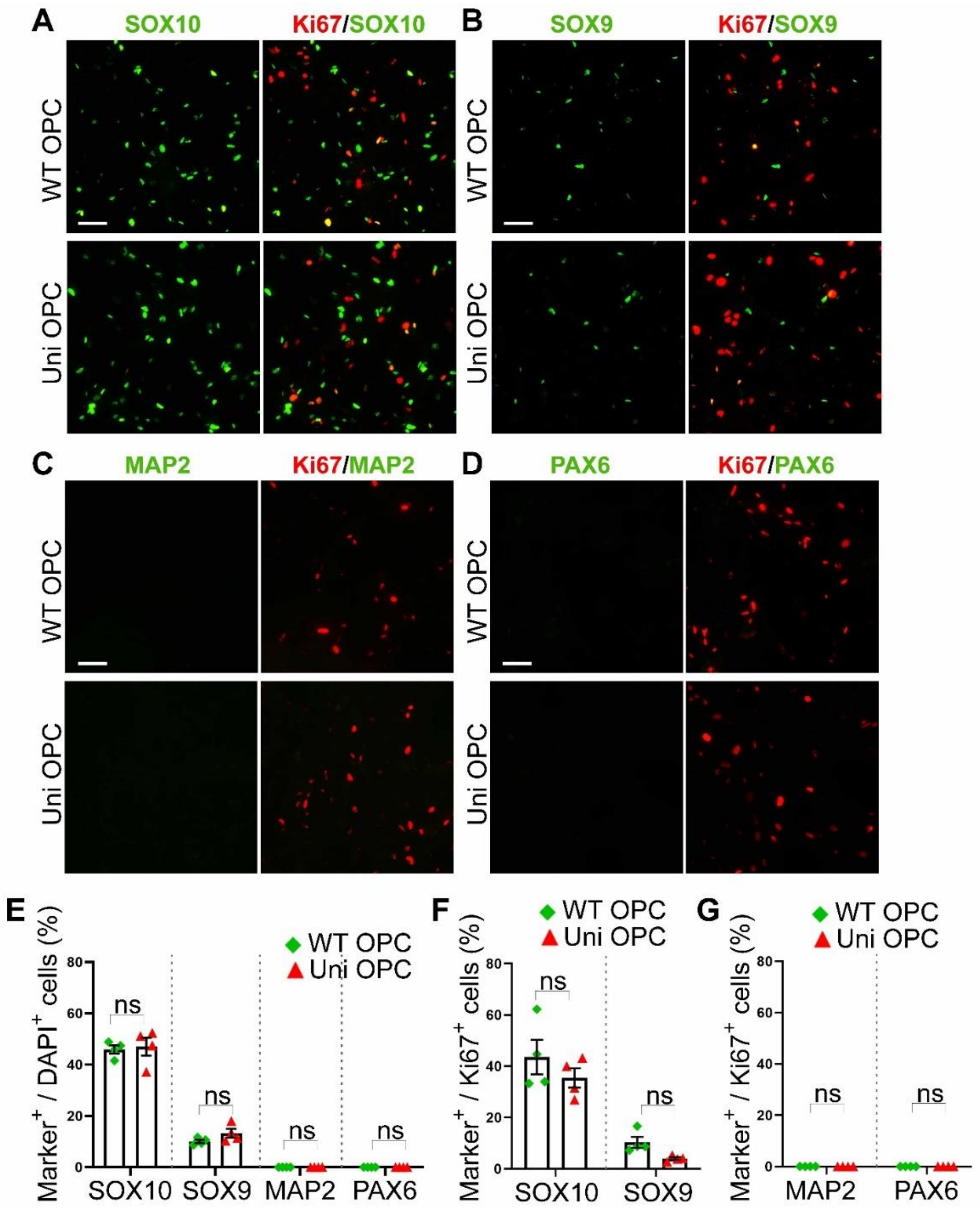

Figure S2. Characterization and proliferation of the WT and uni OPC cell populations in *in vitro*, related to Figure 1. (A-D) The proliferative cells in the OPC population are SOX10$^+$ OPCs, along with some SOX9$^+$ astrocytes. Co-staining Ki67 with the oligodendroglial lineage marker SOX10 (A) the astrocyte marker SOX9 (B), the neuronal marker MAP2 (C), and neural progenitor marker PAX6 (D). (E) The percentage of different neural lineage cells in the OPC population. (F) The percentage of SOX10$^+$ Ki67$^+$ cells or SOX9$^+$ Ki67$^+$ cells in the total Ki67$^+$ cells. (G) The percentage of MAP2$^+$ Ki67$^+$ cells or PAX6$^+$ Ki67$^+$ cells in the total Ki67$^+$ cells. n= 4 images for each marker. Scale bar: 100 µm. ns, not significant by two-way ANOVA followed by Šidák’s multiple comparisons test for panel E-G.

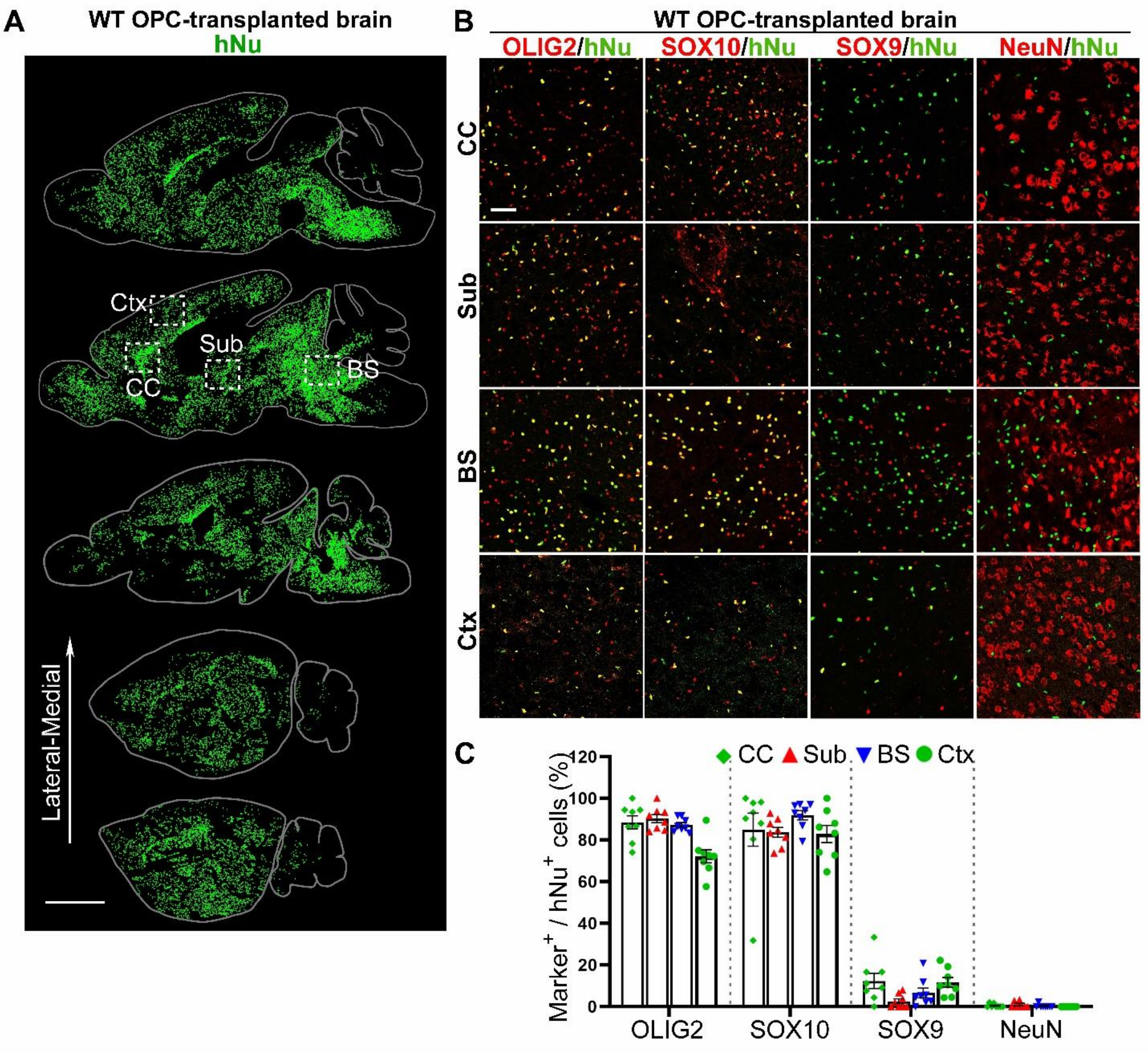

Figure S3. The WT OPCs exhibit widespread distribution after being transplanted into CD (Nur7) mouse brains, related to Figure 2. (A) The WT OPCs migrated and spread widely in the brain six months after transplantation. The dot map of the human nuclear antigen (hNu) staining is shown. Scale bar: 2,000 µm. (B) The WT OPCs gave rise to mostly oligodendroglial lineage cells, a small population of astrocytes and few neurons in the transplanted mouse brains. Six months after transplantation, the WT OPC-transplanted brains were immunostained for hNu and the oligodendroglial lineage markers OLIG2 and SOX10, the astrocyte marker SOX9 and the neuronal marker NeuN. Images from the corpus callosum (CC), the subcortical white matter (Sub), the brain stem (BS) and the cortex (Ctx) were shown. Scale bar: 50 µm. (F) The percentage of hNu^+ and the neural lineage $marker^+$ cells in the WT OPC-transplanted brains. The hNu^+ neural lineage $marker^+$ cells in three regions of the brain, including the corpus callosum (CC), the subcortical white matter (Sub), the brain stem (BS) and the cortex (Ctx), were quantified. n= 8 fields from each region from 4 mice, with 2 images from each region in each mouse brain.

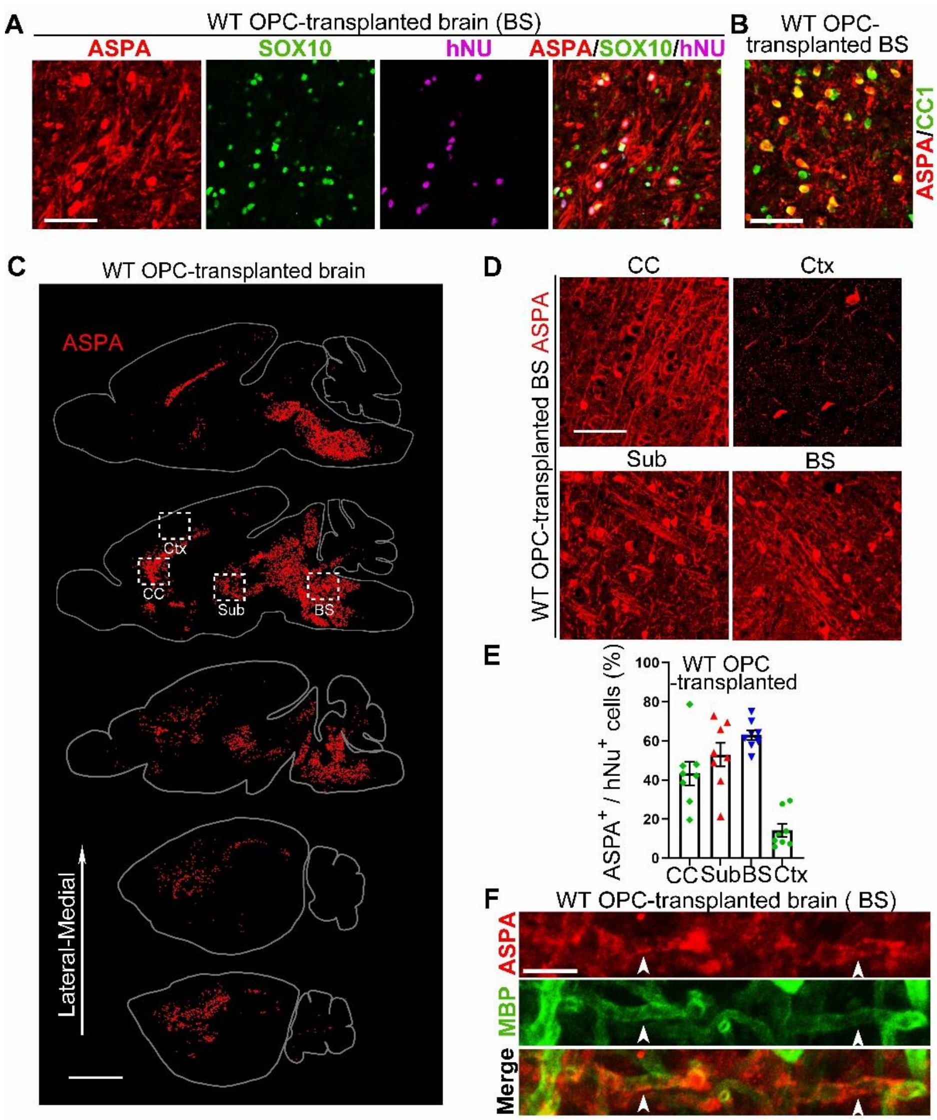

Figure S4. Characterization of WT OPCs after being transplanted into the brains of CD (Nur7) mice, related to Figure 3. (A) Co-expression of ASPA with hNu and the oligodendroglial lineage marker SOX10 in uni OPC-transplanted CD (Nur7) mouse brains. Scale bar: 50 µm. (B) Co-expression of ASPA with the mature oligodendrocyte marker CC1 in the WT OPC-transplanted CD (Nur7) mouse brains. Scale bar: 50 µm. (C) A dot map showing widespread ASPA expression in the white matter track of the brain based on the immunostaining signal of ASPA in the cell body. Scale bar: 2,000 µm. (D) ASPA staining images from the boxed regions in panel C are shown. In addition to cell body expression, ASPA was also expressed in the processes of oligodendrocytes. Shown are images from four regions, including the corpus callosum (CC), the subcortical white matter (Sub), the brain stem (BS) and cortex (Ctx). Scale bar: 50 µm. (E) The percentage of $hNu^{+}ASPA^{+}$ oligodendrocyte cells in the uni OPC-transplanted brains. About half of the human cells matured into oligodendrocytes and expressed the ASPA enzyme in the white matter region. n= 8 fields from each region from 4 mice, with 2 images from each region in each mouse brain. (F) Enlarged images showing the co-localization of ASPA and MBP on the

processes of oligodendrocytes. The arrow heads indicate exampled positions of colocalization. Scale bar: 5 μm.

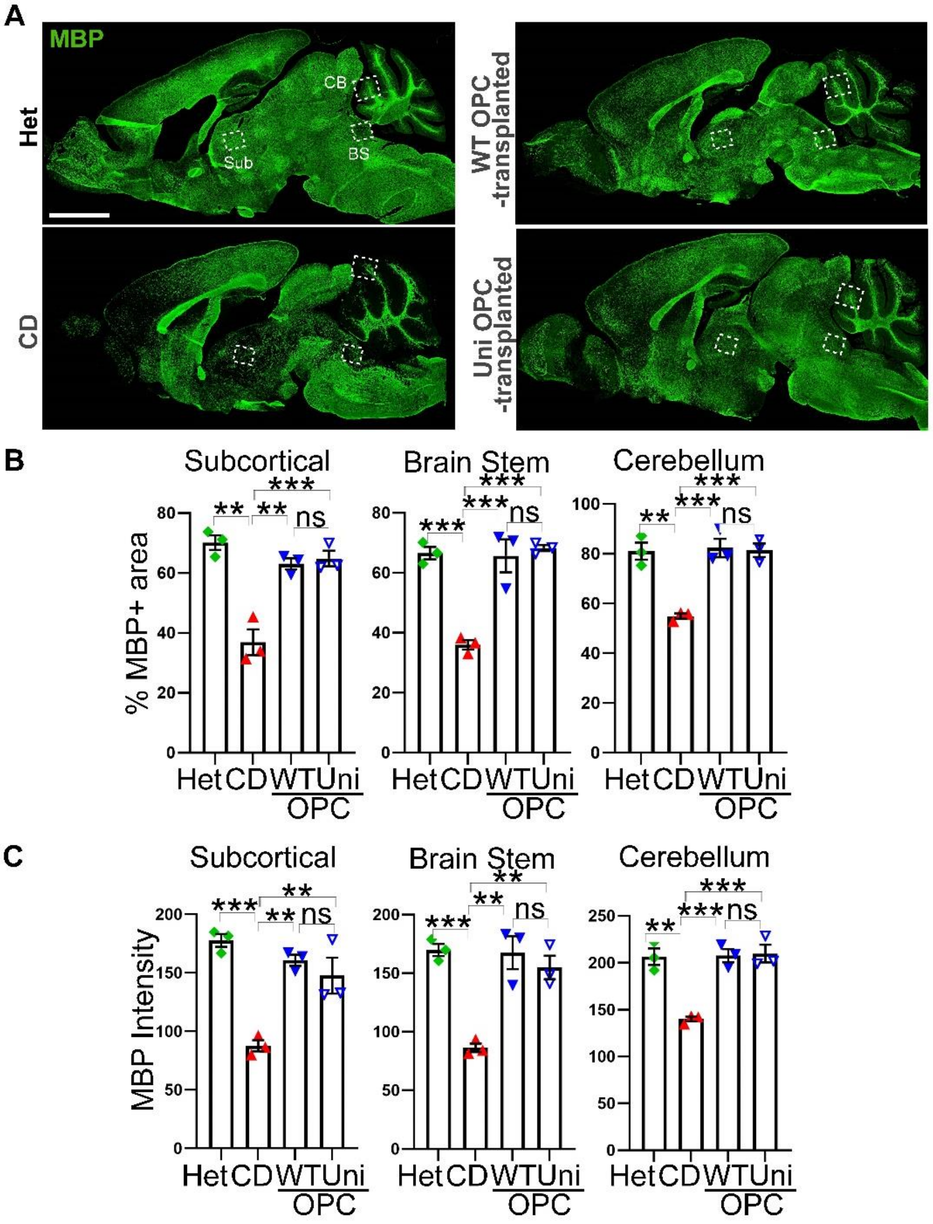

Figure S5. Improved myelination in the WT and uni OPC-transplanted CD (Nur7) mouse brains, related to Figure 4. (A)The whole brain sagittal sections with MBP staining are shown. The boxed regions in the subcortical white matter (Sub), the brain stem (BS) and the cerebellum (CB) are enlarged and shown in Figure 5A. Scale bar: 2,000 µm. (B) Quantification of the MBP$^+$ area in the subcortical white matter, the brain stem, and the white matter of cerebellum. n= 3 mice for each group. (C) Quantification of the MBP intensity in the subcortical white matter, the brain stem and the white matter of the cerebellum. n= 3 mice for each group.

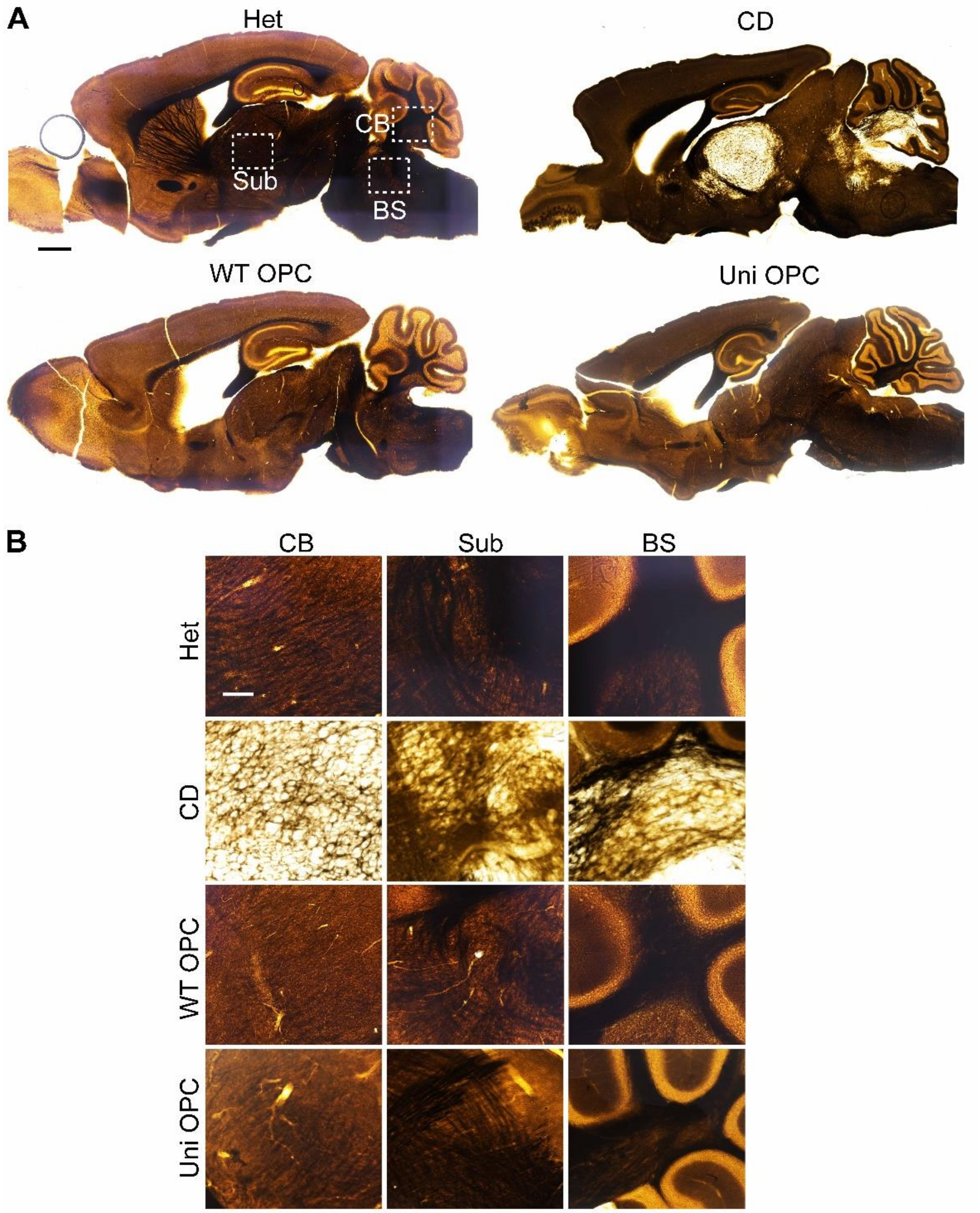

Figure S6. Improved myelination in the WT or universal (uni) OPC-transplanted brains as revealed by the osmium tetroxide (OsO4) staining, related to Figures 4 and 5. (A) Six months after transplantation, the brains of the Het, CD (Nur7), or the WT or uni OPC-transplanted CD (Nur7) mice were analyzed by osmium tetroxide (OsO4) staining. The sagittal whole brain sections are shown. Dramatically reduced myelination was observed in the white matter track of CD (nur7) mouse brains, whereas the demyelination was rescued in the WT or uni OPC-transplanted mouse brains. Scale bar: 2,000 μm (B) The enlarged images from the boxed regions in panel A are shown. Scale bar: 200 μm

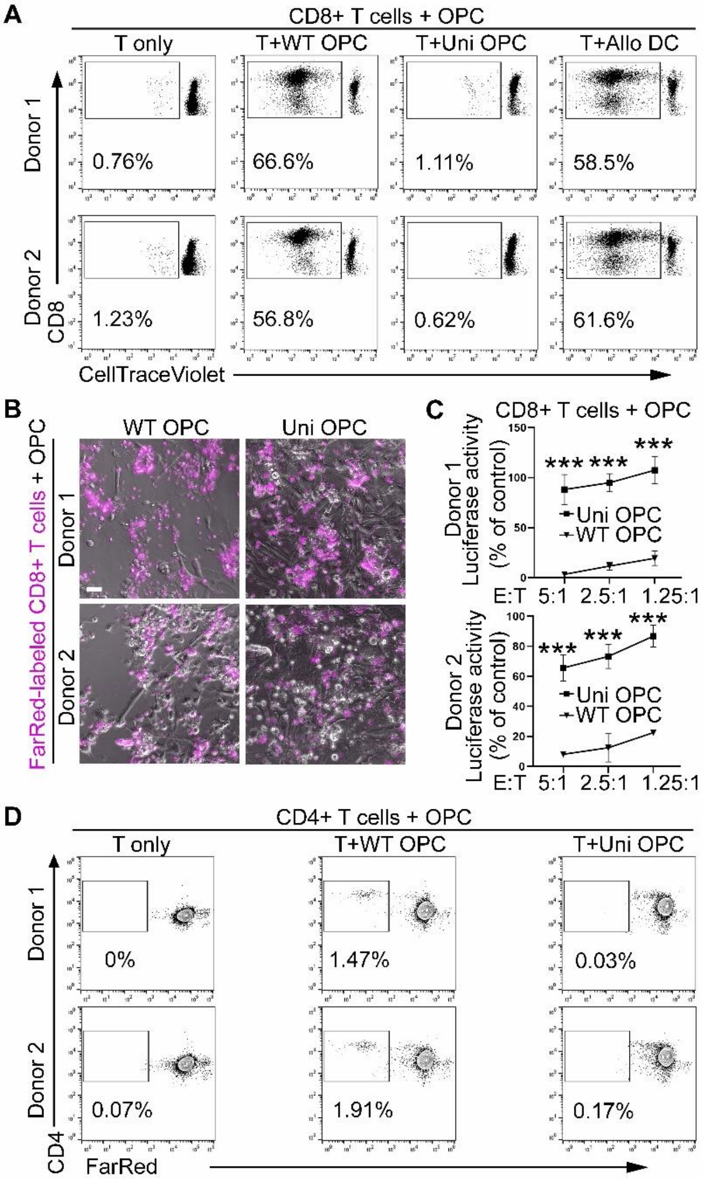

Figure S7. The universal (uni) OPCs can evade the response from $CD8^+$ and $CD4^+$ T cells from different donors, related to Figure 6. (A) The WT but not the uni OPCs stimulated the expansion of the $CD8^+$ T cells. The CellTrace proliferation assay was performed to detect proliferative $CD8^+$ T cells from different donors in response to the WT or the uni OPCs. The allogenic dendritic cells (DC) were included as the positive control, and the $CD8^+$ T cell only as the negative control. (B, C) The uni OPCs escaped the lytic activity of the $CD8^+$ T cells largely. The WT OPCs were included as a control. The images (B) and the luciferase activity (C) of theWT or uni OPCs after co-culturing with the reactive $CD8^+$ T cells from different donors. Scale bar: 100 µm. (D) The WT but not the uni OPCs stimulated the expansion of the $CD4^+$ T cells from different donors. The CellTrace proliferation assay was performed to detect proliferative $CD4^+$ T cells from different donors in response to the WT or the uni OPCs. ***p<0.001 by two-way ANOVA followed by Benferroni's multiple comparisons test for panel C.

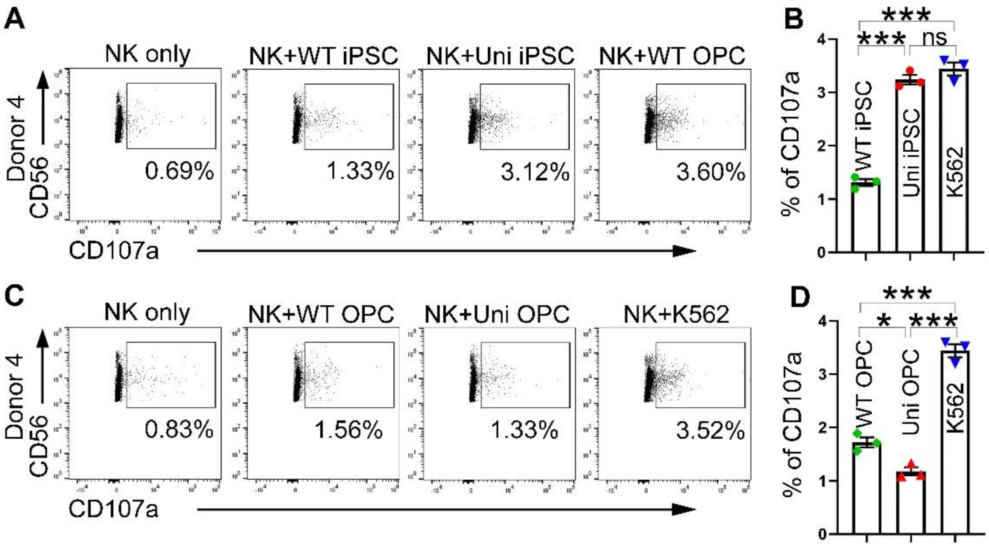

Figure S8. The universal (uni) OPCs can evade NK cell response in *in vitro*, whereas uni iPSC cannot for additional donor, related Figure 7. (A, B) The uni iPSCs were not able to evade the NK response in additional donor. The WT or uni iPSCs were co-cultured with NK cells isolated from allogenic PBMC. The CD107a degranulation assay to assess NK cell activation is shown in panel A. The K562 cells were included as the positive control and the NK cells only as the negative control. Quantification from three experiments is shown in panel B. n=3 biological repeats. (C, D) The uni OPCs were able to evade the response from NK cells of different donors. The WT or uni OPCs were co-cultured with NK cells isolated from allogenic PBMC. The CD107a degranulation assay are shown in panel C and D. The K562 cells were included as the positive control and the NK cells only as the negative control. Quantification from three experiments for donor 4 is shown in panel F. NK cells exhibited similar degranulation activity in response to the WT or the uni OPCs. n=3 biological repeats. Error bars are SE of the mean. ns, not significant, *p<0.05, **p<0.01, and ***p<0.001 by one-way ANOVA followed by Tukey's multiple comparisons test for panel B and D.

Table. S1 Antibody list

Antibodies	SOURCE	IDENTIFIER
Mouse monoclonal IgM anti-O4	Sigma-Aldrich	Cat# O7139
Rabbit polyclonal anti-OLIG2	Millipore Sigma	Cat# AB9610
Goat polyclonal anti-SOX10	R&D	Cat# AF2864
Chicken polyclonal anti-ASPA	Abcam	Cat# ab5392
Rabbit polyclonal anti-PAX6	Biolegend	Cat# 901301
Mouse monoclonal anti-human nuclear antigen antibody [235-1], hNu	Abcam	Cat# Ab191181
Goat polyclonal anti-SOX9	R&D	Cat# AF3075
Rabbit polyclonal anti-NeuN	GeneTex	Cat# GTX16208
Mouse monoclonal anti-CC1 (APC)	Millipore Sigma	Cat# MABC200
Rabbit polyclonal anti-ASPA	Abcam	Cat# ab97454
Rat monocolonal anti-MBP	Millipore Sigma	Cat# MAB386
Rabbit monoclonal anti-Ki67	ThermoFisher Scientific	Cat# RM-9106-S0
PE Mouse anti-CD140a	BD Biosciences	Cat # 556002
APC Mouse Anti-Human HLA-ABC, HLA-I	BD Biosciences	Cat# 562006
Alexa Fluor® 647 Mouse Anti-Human HLA-DR, DP, DQ; HLA-II	BD Biosciences	Cat# 563591
FITC Mouse Anti-Human CD45	BD Biosciences	Cat# 555482
PE Mouse Anti-Human CD45	BD Biosciences	Cat# 555483
PE Rat Anti-Mouse CD45	BD Biosciences	Cat# 553081
PE-Cy5 Mouse Anti-Human CD3	BD Biosciences	Cat# 555341
PE Mouse Anti-Human CD8	BD Biosciences	Cat# 561949
FITC Mouse Anti-Human CD4	BD Biosciences	Cat# 561005
APC Mouse Anti-Human CD56	BD Biosciences	Cat# 555518
FITC Mouse Anti-Human CD107a	BD Biosciences	Cat# 555800
PerCP-Cy™5.5 Mouse Anti-Human CD47	BD Biosciences	Cat# 561261

Cy3 Donkey Anti-Mouse IgM	Jackson Immuno	Cat# 715-165-020
Alexa Fluor® 647 Donkey Anti-Rabbit IgG	Jackson Immuno	Cat# 711-605-152
Alexa Fluor® 488 Donkey Anti-Goat IgG	Jackson Immuno	Cat# 705-545-147
Alexa Fluor® 647 Donkey Anti-Mouse IgG	Jackson Immuno	Cat# 715-605-151
Cy3 Donkey Anti-Rabbit IgG	Jackson Immuno	Cat# 711-165-152
Cy3 Donkey Anti-Rat IgG	Jackson Immuno	Cat# 712-165-150
Alexa Fluor® 647 Donkey Anti-Rat IgG	Jackson Immuno	Cat# 712-605-153
PE Mouse IgG2a, κ Isotype Control	BD Biosciences	Cat# 555574
APC Mouse IgG1 κ Isotype Control	BD Biosciences	Cat# 554681
Alexa Fluor® 647 Mouse IgG2a, κ Isotype Control	BD Biosciences	Cat# 565357
FITC Mouse IgG1, κ Isotype Control	BD Biosciences	Cat# 555748
PE Mouse IgG1, κ Isotype Control	BD Biosciences	Cat# 554680
PerCP-Cy5.5 Mouse IgG1, κ Isotype Control	BD Biosciences	Cat# 347212
